# Emotion-Regulation Difficulties and Skills in the Cross-Sectional Association Between Thyroid Function and Antenatal Distress in Hypothyroid Pregnancy

**DOI:** 10.3390/healthcare14172700

**Published:** 2026-08-24

**Authors:** Resat Misirlioglu, Filiz Yarsilikal Guleroglu, Ali Cetin

**Affiliations:** Department of Obstetrics and Gynecology, Haseki Training and Research Hospital, University of Health Sciences, 34265 Istanbul, Türkiye

**Keywords:** hypothyroidism, pregnancy complications, cross-sectional studies, thyroxine, thyrotropin, emotional regulation, antenatal depressive symptoms, pregnancy-related anxiety, Türkiye

## Abstract

Background/Objectives: Thyroid dysfunction and emotion-regulation difficulties are each linked to antenatal distress but are rarely measured in the same women. We examined whether emotion regulation accounts for the cross-sectional association between thyroid biochemistry and antenatal distress in hypothyroid pregnancy. Methods: In this single-center cross-sectional study, 360 pregnant women with hypothyroidism completed measures of emotion-regulation difficulties and skills and four distress measures at one antenatal visit, alongside thyroid testing. Exposures were standardized log thyroid-stimulating hormone (TSH) and free thyroxine (free T4); the outcome was a composite distress score. Parallel two-mediator models with covariate adjustment were fitted, and bias-corrected bootstrapping was used to estimate the indirect associations. Results: Higher log TSH (total association 0.189, 95% confidence interval [CI] 0.108 to 0.272) and lower free T4 (−0.200, 95% CI −0.278 to −0.120) were associated with greater distress. Most of each association was statistically accounted for by emotion-regulation difficulties and skills; direct associations included zero. Apparent route-specific moderation by gestational week and by thyroid autoimmunity was not supported, because the two indices did not differ. Conclusions: Measurement at a single visit leaves temporal ordering unidentified, so these findings decompose a cross-sectional association rather than demonstrate causal mediation; within that constraint they support assessing emotion regulation alongside thyroid monitoring and depression screening.

## 1. Introduction

Hypothyroidism is the most common thyroid disorder of pregnancy and, in iodine-sufficient and mildly iodine-deficient populations, is most often due to chronic autoimmune thyroiditis. Both overt and subclinical hypothyroidism have been associated with depressive and anxiety symptoms and with clinical depression in population-based synthesis [1], and thyroid hormone acts on limbic and prefrontal circuits that support mood regulation, providing a plausible biological substrate for this association [2,3,4]. The clinical picture is complicated by symptom overlap: fatigue, poor concentration, low mood, weight change and sleep disturbance are features of both hypothyroidism and pregnancy itself, so that hypothyroid women may experience an emotional burden that is neither fully recognized nor fully attributable. Thyroid autoimmunity carries additional risk: anti-thyroid-peroxidase antibodies in gestation predict subsequent perinatal depressive symptoms independently of biochemical thyroid status in several cohorts [5,6], and more recent work confirms both an increased risk of postpartum depression with thyroid autoimmunity and a dose-dependent relationship between antibody concentration and maternal thyroid function [7,8]. Most studies, however, infer a biological mechanism from the mere co-occurrence of thyroid abnormality and symptom burden. The psychological processes that might accompany a thyroid–distress association have rarely been measured in the same women.

The present study is confined to hypothyroidism; women with Graves disease or thyrotoxicosis were not eligible.

Emotion regulation is a plausible candidate for such a process. As a transdiagnostic construct governing the onset and maintenance of both depression and anxiety, it operates through overlapping strategies that act as risk and protective factors across the two conditions [9,10]. Two related but non-redundant facets are usually distinguished: difficulties in regulating emotion, indexed by measures derived from the difficulties-in-emotion-regulation framework [11], and the availability of adaptive skills such as awareness, acceptance, tolerance, and the capacity to modify emotional responses. Treating these facets as opposite ends of a single scale obscures their distinct contributions. Emotion regulation in pregnancy has itself been identified as an under-researched yet modifiable target for perinatal mental-health screening and intervention [12].

Pregnancy, with its pronounced endocrine and psychological change, is a useful setting in which to examine whether regulatory processes accompany, and statistically account for, the association between a biological signal such as thyroid status and emotional distress. Hypothyroid pregnancy is especially pertinent: these women are already under surveillance for biochemical control, and any regulatory dimension accompanying that biochemistry would bear directly on how their antenatal mental-health needs are recognized and met.

The local context is relevant to both halves of this question. In Turkish antenatal populations, depressive symptoms above conventional screening thresholds have been reported in 14.6% to 27.5% of pregnant women, the range reflecting differences in instrument and cut-off [13,14], and pregnancy-related anxiety is at least as common, at 29.6% in the largest Turkish perinatal cohort [13]; these figures bracket the 23.6% of our cohort who screened positive on the Edinburgh Postnatal Depression Scale. Reported prevalences of hypothyroidism in Turkish pregnancy vary widely with the threshold applied: in a Turkish universal-screening cohort of 1416 women, subclinical hypothyroidism was 22.3% at a thyroid-stimulating hormone cut-off of 2.5 mIU/L, but total hypothyroidism was only 2.9% at a cut-off of 4.0 mIU/L [15]—an inconsistency of definition, not of biology, to which we return in the Discussion.

We therefore examined, in pregnant women with hypothyroidism, whether difficulties in emotion regulation and adaptive regulation skills account for the cross-sectional association between thyroid biochemistry and antenatal distress. We deliberately kept standardized log TSH and standardized free T4 as separate primary exposures rather than collapsing them into one index, since a single value can correspond to clinically distinct states. We also examined whether any indirect association shifted across gestation or with thyroid autoimmunity. Given the cross-sectional design, we treat the analysis throughout as a decomposition of association, not as a test of causal mediation.

## 2. Materials and Methods

### 2.1. Study Design and Participants

This single-center, cross-sectional, observational study was carried out in the tertiary perinatology unit of the Haseki Training and Research Hospital, University of Health Sciences, Istanbul, Türkiye, between December 2023 and February 2026, and is reported according to the Strengthening the Reporting of Observational Studies in Epidemiology (STROBE) statement [16]. Each woman contributed data from a single antenatal visit at which thyroid biochemistry and psychological measures were obtained together, so the two sets of measures refer to the same time point. Gestational age was assigned from the first day of the last menstrual period (LMP) when this agreed with first-trimester crown–rump length (CRL) ultrasonography; when menstrual and first-trimester ultrasound dating differed by more than seven days, gestational age was reassigned from the ultrasound examination, following contemporary obstetric dating recommendations [17], and all subsequent follow-up and analyses used the revised age. Because the cohort was managed for hypothyroidism at different treatment stages, current biochemistry reflected a mix of treated, subclinical, and overt states, not a uniform disease severity.

Inclusion criteria were as follows: (i) age 18 years or older; (ii) singleton pregnancy; (iii) a documented diagnosis of hypothyroidism, defined biochemically according to pregnancy-specific guidance [2] as an elevated serum thyroid-stimulating hormone concentration interpreted against a pregnancy-appropriate upper reference limit, with or without a low free thyroxine concentration, or an established diagnosis of hypothyroidism currently treated with levothyroxine; (iv) attendance for routine antenatal care at the study unit; (v) ability to read and complete the self-report questionnaires in Turkish; and (vi) provision of written informed consent. Eligibility was based on a documented historical diagnosis of thyroid dysfunction recorded in the medical record; current levothyroxine treatment, biochemical thyroid dysfunction at the index antenatal visit and thyroid-antibody positivity were not required for inclusion.

Exclusion criteria were as follows: (i) multiple gestation; (ii) known Graves disease or thyrotoxicosis; (iii) a major fetal anomaly identified before assessment; (iv) current or past primary psychotic or bipolar disorder; (v) current psychotropic pharmacotherapy; (vi) an acute condition requiring hospitalization at the time of assessment; and (vii) inability to complete the questionnaires because of literacy or language barriers.

Some conditions were not excluded. Coexisting conditions that are themselves associated with antenatal distress, in particular gestational diabetes and unplanned pregnancy, were deliberately not treated as exclusions. The quantity of interest is the association between thyroid biochemistry and distress in the hypothyroid antenatal population to whom any screening recommendation would apply, not in a comorbidity-free subset of it, and both conditions are confounders to be adjusted for, not competing exposures to be removed. Both were therefore retained, entered as covariates in every model (Section 2.4), and examined directly by re-estimating the primary models with each group excluded (Section 3.5). Excluding them at the outset would have produced a cohort unrepresentative of routine practice without removing the confounding, since the same reasoning would require excluding women with low income, limited social support or nulliparity, who together account for a large share of the cohort.

The diagnostic basis was as follows. Hypothyroidism was diagnosed biochemically, in accordance with pregnancy-specific guidance [2,18], from serum thyroid-stimulating hormone interpreted against a pregnancy-appropriate reference limit, together with free thyroxine where available. Thyroid ultrasonography was not used as a diagnostic criterion, since ultrasonography characterizes thyroid morphology and not thyroid function and is not required for the diagnosis of hypothyroidism by any current guideline: a sonographically normal gland is fully compatible with overt hypothyroidism, and nodular or goitrous glands are frequently euthyroid. In this unit, thyroid ultrasonography is performed on clinical indication—palpable goiter, a palpable or incidentally detected nodule, or surveillance of a previously documented nodule during pregnancy, with ultrasonography being the only imaging modality available in pregnancy since radionuclide imaging is contraindicated—rather than as part of routine antenatal thyroid assessment. Its absence as a routine investigation is addressed in the Limitations, with specific reference to seronegative autoimmune thyroiditis.

Supervision of care was carried out as follows. Thyroid-function monitoring and referral for endocrinology consultation were determined by the treating obstetrician according to clinical findings and routine care and not by a study-mandated follow-up protocol. Because the intensity of endocrine supervision was therefore not uniform across the cohort, thyroid biochemistry at the index visit reflects heterogeneous monitoring and titration practice; we note this as a limitation. During the index pregnancy, the median number of thyroid-function measurements recorded was 3 (interquartile range 2 to 5), and 159 women (44.2%) attended at least one endocrinology consultation.

No restricted gestational-age window was applied. Gestational age was a prespecified moderator of the indirect associations, and its wide distribution (6.6 to 38.8 weeks) was a design requirement, not an incidental feature: estimation of the index of moderated mediation and of the Johnson–Neyman region of significance across gestation is possible only when gestational age varies substantially between participants, so a restricted window would have made the second of our two research questions unanswerable. Gestational age also entered every model as a covariate, so that the reported thyroid–distress associations are adjusted for gestational age as a main effect in addition to being examined as a moderator, and thyroid and psychological maesures were also summarized by trimester.

The timing of the thyroid diagnosis was determined from the earliest available diagnosis, endocrinology assessment, or levothyroxine-prescription record. It was classified as pre-pregnancy when documented before the estimated date of conception, as pregnancy-onset when first documented during the index pregnancy in the absence of an earlier record, and as unclassifiable when the available records did not permit reliable temporal classification. A diagnosis made at another institution before conception was classified as pre-pregnancy, and first attendance at this unit during pregnancy was not by itself treated as pregnancy-onset disease. Hypothyroidism had been diagnosed before the index pregnancy in 174 women (48.3%), was first documented during the index pregnancy in 161 (44.7%), and could not be reliably classified in 25 (6.9%).

Sampling was consecutive, not by convenience: during the recruitment period, every woman attending routine antenatal care at the unit with a documented diagnosis of hypothyroidism was screened for eligibility, and every eligible woman was invited to participate. No quota, matching or stratified selection was applied, and no woman was selected or passed over on the basis of thyroid biochemistry, symptom burden or psychological status.

### 2.2. Thyroid Measures

Serum thyroid-stimulating hormone (TSH), free thyroxine (free T4), free triiodothyronine (free T3), anti-thyroid-peroxidase antibody, and anti-thyroglobulin antibody were measured from venous blood drawn at the same antenatal visit as the psychological assessment. All analytes were measured using an Atellica IM Analyzer (Siemens Healthineers, Erlangen, Germany) with the manufacturer’s chemiluminescent immunoassays and reagents. Throughout the study, testing was confined to a single accredited hospital central laboratory using one analytical platform, a standardized internal quality control, routine daily calibration, and the manufacturer’s external quality-assurance protocols, which limited analytical variability between participants.

Recruitment spanned 26 months. Throughout this period all analytes were measured on the same analyzer using the same assay methods and calibrator lineage; reagent and calibrator lots changed in the normal course of laboratory operation, and each new lot was verified against internal quality-control material before release, with control results reviewed for shift and drift under the laboratory’s accredited quality-management system. The laboratory confirmed that no change of assay method, in manufacturer decision threshold, in calibration standardization or in reporting unit occurred during the study period and that no migration from another immunoassay system took place.

TSH was measured with a third-generation TSH3-UL two-site chemiluminescent assay (measuring interval 0.008 to 150 mIU/L; within-laboratory coefficient of variation [CV] up to approximately 3.7% across the clinical range; traceable to the World Health Organization (WHO) Third International Standard for Thyroid-Stimulating Hormone, Human, for Immunoassay (IRP 81/565)). Free T4 and free T3 were measured with competitive direct chemiluminescent assays (free T4 measuring interval 0.1 to 12.0 ng/dL, within-laboratory CV up to approximately 6.8%; free T3 measuring interval 0.20 to 20.0 pg/mL, within-laboratory CV approximately 1 to 2% within the reference range). Anti-thyroid-peroxidase antibody was measured with a competitive chemiluminescent assay (measuring interval 28 to 1300 U/mL; within-laboratory CV up to approximately 7.4%; traceable to WHO reference preparation MRC 66/387) and anti-thyroglobulin antibody with a one-step analyte-bridging acridinium-ester chemiluminescent assay (measuring interval 1.3 to 1000 IU/mL; within-laboratory CV up to approximately 3.7%; traceable to WHO reference preparation NIBSC 65/093). The reported coefficients of variation are the manufacturer’s within-laboratory precision estimates.

Hypothyroidism was defined from the documented clinical diagnosis together with pregnancy-specific thyroid guidance [2,18]. The manufacturer’s adult non-pregnant reference intervals for these assays were 0.55 to 4.78 mIU/L for TSH, 0.89 to 1.76 ng/dL for free T4 and 2.3 to 4.2 pg/mL for free T3, with anti-thyroglobulin positivity defined at 4.5 IU/mL. Anti-thyroid-peroxidase positivity, the primary autoimmune marker in all analyses, was defined at the manufacturer decision threshold of 60 U/mL. Trimester-specific pregnancy reference intervals were not provided by the manufacturer for these assays; TSH was therefore interpreted following guideline-based practice, using a TSH upper reference limit of approximately 4.0 mIU/L, while TSH was retained as a continuous standardized exposure in all primary models. Low free T4 was defined by the assay-specific laboratory lower reference limit, and we also report a descriptive clinical threshold of free T4 below 0.80 ng/dL. Because free T4 immunoassay values are platform-dependent in pregnancy, free T4 was analyzed primarily as a continuous standardized marker rather than as a dichotomous diagnostic category, and dichotomous thyroid thresholds were used only for descriptive clinical reporting. The manufacturer non-pregnant intervals were used for analytical assay verification, whereas clinical interpretation during pregnancy followed pregnancy-specific guideline recommendations.

TSH was right-skewed and was natural-log transformed before standardization; free T4 and free T3 were standardized in their original units (ng/dL and pg/mL). All index-visit thyroid measurements were performed on the same Atellica IM platform throughout the study period; no migration from another immunoassay system occurred, and although routine reagent and calibrator lots changed, the assay method, the manufacturer decision threshold, the calibration standardization and the reporting unit did not. Anti-thyroid-peroxidase concentrations are reported in U/mL and anti-thyroglobulin concentrations in IU/mL, as specified by the respective package inserts. Thyroid autoimmunity was defined a priori for the primary analysis as an anti-thyroid-peroxidase concentration at or above the manufacturer decision threshold of 60 U/mL. No locally derived decision threshold was in use, and no local threshold-derivation study, reference population or percentile document exists. For the Atellica IM aTPO assay, the measuring interval is 28 to 1300 U/mL, the limit of blank is 13 U/mL and the limit of detection is 20 U/mL. Quantitative reporting of patient results below 28 U/mL is not validated, and the numeric values below that limit present in the research export were therefore not treated as analytically validated quantitative measurements. In this sample 243 results (67.5%) lay below 28 U/mL, 212 (58.9%) below the limit of detection and 153 (42.5%) below the limit of blank. Because concentrations in that region are not quantitative, thyroid autoimmunity was modeled as a binary variable, and analyses using the natural-log-transformed continuous concentration were retained only as sensitivity analyses. The highest observed anti-thyroid-peroxidase value was 1000 U/mL; because the measuring interval extends to 1300 U/mL this is not an assay ceiling, and the sensitivity analysis previously described as removing an assay-ceiling value is reported here as removing the single highest observed value.

### 2.3. Emotion Regulation and Distress Measures

Difficulties in emotion regulation were assessed with the 16-item Difficulties in Emotion Regulation Scale (DERS-16), a brief measure with strong internal consistency and construct validity [19]. Adaptive regulation skills were assessed with the Emotion Regulation Skills Questionnaire (ERSQ), which captures awareness, clarity, understanding, bodily-sensation processing, acceptance, tolerance, readiness to confront, self-support, and modification of emotions [20,21]. Because the DERS-16 indexes difficulties and the ERSQ indexes skills, we treated them as complementary dimensions, not as opposite poles of one construct.

Antenatal distress was captured by four validated instruments. Depressive symptom burden was measured with the 10-item Edinburgh Postnatal Depression Scale (EPDS), which asks respondents to rate their experience over the preceding seven days and includes an item on self-harm ideation [22]; despite its name, which reflects the postnatal sample in which it was originally developed, the instrument has been validated for antenatal administration and is widely applied across pregnancy, where it is also referred to as the Edinburgh Depression Scale [23]. The EPDS is a screening measure of current depressive symptom burden and is not a diagnostic instrument, so that a score at or above a cut-off identifies a woman as screen-positive for probable depression and indicates a need for further clinical assessment, whereas diagnostic status can be established only by a structured or clinical diagnostic interview [24], and no diagnostic interview was administered in this study. The revised Pregnancy-Related Anxiety Questionnaire (PRAQ-R2) measured pregnancy-specific anxiety across parity groups [25]; the Pregnancy-related Anxiety Scale (PrAS) gave a multidimensional index of pregnancy-related worry [26]; and the Cambridge Worry Scale (CWS) measured the content and extent of antenatal worries and has been validated in Turkish pregnant women [27,28]. We treated these four measures as indicators of a single antenatal distress dimension. A confirmatory factor model confirmed that they loaded on one common factor, and the outcome entered into the association models was a standardized composite distress score built from the four indicators. We used this composite rather than a latent-variable score so that the parallel two-mediator paths could be estimated within the bias-corrected bootstrap framework described below, with the confirmatory factor model providing the unidimensionality that justifies a single-score outcome. All self-report instruments were administered in validated Turkish adaptations: the DERS-16 [29], the ERSQ [30], the EPDS [31], the PRAQ-R2 [32], the PrAS [33], and the Cambridge Worry Scale [28].

The Turkish adaptation of the EPDS was validated among women within their first postpartum year [31]; to our knowledge a formal Turkish-language validation in an antenatal sample has not been published. The English-language instrument has been validated antenatally, where trimester-specific optimal cut-offs of 11 in the first trimester and 10 in the second and third have been reported [23], and an individual-participant-data meta-analysis has quantified its accuracy and confirmed that screen-positive status overestimates diagnosed major depression [24]; a systematic review has separately shown that antepartum and postpartum psychometric properties are not interchangeable [34]. The EPDS is used routinely across pregnancy in Turkish perinatal practice and research, but the psychometric properties and the optimal cut-off of the Turkish version in pregnancy have not been established. We therefore used the EPDS total score as a continuous indicator of depressive symptom burden in all primary analyses and applied the postpartum-derived threshold of 13 only for descriptive reporting and safety referral. In this antenatal sample the Turkish EPDS showed acceptable internal consistency (α = 0.753).

A safety protocol was in place: women who screened positive on the EPDS at a total score of 13 or above, who endorsed the EPDS self-harm item, or who reached a severe pregnancy-anxiety threshold were referred for clinical assessment per study protocol. Referral was triggered by a screening result, not by a diagnosis; the study did not establish diagnostic status for any participant. Post-referral clinical outcomes were not captured systematically in the analytic dataset, which we note as a limitation.

#### Questionnaire Administration

All instruments were completed as one self-administered packet during a single session at the index antenatal visit in a fixed order (DERS-16, ERSQ, EPDS, PRAQ-R2, PrAS, and CWS), taking a median of 28 min (interquartile range 24 to 33; approximate range 18 to 46) in a quiet private interview room adjacent to the perinatology clinic. Participants completed the packet alone in the room; research staff were available outside if needed but were not present during completion, and returned packets were checked afterwards for missing items and for the safety items. The order was not randomized or counterbalanced. Questionnaires were completed before any thyroid result from that visit was available to or communicated with the participant so that no woman completed the instruments in the knowledge of her index-visit biochemistry, although all participants were necessarily aware of their pre-existing thyroid diagnosis. Participants were assured of confidentiality and told that participation would not affect their clinical care; individual responses were not entered into the clinical record except where the safety protocol was triggered, and no validated social-desirability scale was administered. Women unable to complete written Turkish-language instruments were excluded at screening (*n* = 6). Participants whose packets remained incomplete were excluded after enrollment (*n* = 11, together with those whose same-visit thyroid biochemistry could not be linked), which is why no missing values remained on the analyzed variables.

### 2.4. Covariates

Models were adjusted for gestational age at assessment, maternal age, pre-pregnancy body mass index, parity, education, income, social support, current smoking, planned pregnancy, gestational diabetes, chronic hypertension, and levothyroxine use. Gestational age was entered as a main effect in every equation of every model and, in the moderation analyses, in the relevant product terms as well. Gestational diabetes and chronic hypertension were prespecified covariates but were infrequent in this cohort (15 and four women, respectively); their inclusion reflects prespecification and not effective confounding control, and residual confounding by these conditions cannot be excluded. Gestational diabetes and chronic hypertension were recorded as present or absent at the index antenatal visit so that women assessed before the standard 24-to-28-week screening window could not yet have been classified; the prevalence of gestational diabetes reported here should be read as prevalence at the time of assessment, not as cumulative pregnancy incidence, and is therefore underestimated by design. Prior psychiatric history, one of the strongest predictors of antenatal distress, was unavailable in the dataset; we treat its absence as a source of residual confounding, not as a controlled covariate.

For women receiving levothyroxine, the prescribed daily dose at the index visit was abstracted from the hospital prescription record for this revision. A reliable current dose was retrievable for 168 of the 209 treated women (80.4%), with a median of 62.5 µg per day (interquartile range 50 to 88). Dose could not be established reliably in 41 women (19.6%) because the prescription had been issued at another institution, because the medication list recorded the drug without a dose, or because an earlier dose had been overwritten by a later one without a retained history. Treatment initiation date, the history of dose adjustment and medication adherence could not be established with sufficient reliability for any participant. Because dose was unavailable for approximately one in five treated women and was abstracted only for this revision, it was not entered into the association models. A dose-adjusted model would have been fitted in a subset defined by the completeness of the prescription record, not by any clinical criterion, would not have been comparable with the prespecified primary specification, and would still have carried no information on how long treatment had been given or whether it was taken. Dose is therefore reported descriptively, and the absence of duration and adherence data remains a limitation.

### 2.5. Statistical Analysis

We state the target quantity and its assumptions explicitly. Because exposures, mediators and outcome were measured at a single visit, the quantities we estimate are the components of a linear decomposition of an observed cross-sectional association: the portion of the exposure–outcome association that is statistically accounted for by the two regulatory dimensions and the portion that is not. Reading these quantities as natural direct and indirect effects would further require no unmeasured exposure–outcome confounding, no unmeasured mediator–outcome confounding, no unmeasured exposure–mediator confounding, no mediator–outcome confounder affected by the exposure, and correct temporal ordering of exposure, mediator, and outcome. The final assumption is not merely untested here but unidentifiable by design, and the second is implausible given that prior psychiatric history was unavailable. We therefore report all quantities as associations and use total association in place of total effect throughout, and we make no claim about the direction of influence between thyroid biochemistry, emotion regulation and distress. Because the DERS-16 and ERSQ are related but distinct, the residual covariance between the mediators was freely estimated rather than fixed at zero. We fitted separate models for standardized log TSH and standardized free T4 and analyzed standardized free T3 as a sensitivity exposure.

The choice of methods followed from the structure of the question. Because the outcome, the mediators and the exposures were continuous and approximately symmetric after transformation, associations were modeled with ordinary least squares within a path framework in preference to categorical or rank-based methods, which would have discarded information; TSH and both antibody titers were natural-log transformed before standardization because their raw distributions were strongly right-skewed. Inference for the indirect associations was based on the bias-corrected bootstrap with 5000 resamples in place of the Sobel test, because the sampling distribution of a product of regression coefficients is skewed and the normal approximation underlying the Sobel test is poorly calibrated at these sample sizes [35]; the bootstrap makes no distributional assumption about the product and preserves the dependence between the constituent coefficients. Moderation was assessed with the index of moderated mediation instead of by comparing subgroup-specific indirect associations, because observing that one subgroup estimate reaches significance and another does not is not a test of difference; the index provides a single quantity with a confidence interval, and we tested the difference between the two route-specific indices within the same bootstrap resamples so that the covariance between them is respected. Because four moderation indices were examined, the Benjamini–Hochberg false-discovery-rate procedure [36] was applied within that family and, separately, to the primary indirect associations; the false-discovery-rate approach was preferred to family-wise correction because the moderation analyses are exploratory in intent. Both moderators were standardized before forming the product terms, so each index of moderated mediation was expressed per standard deviation of the moderator. Bootstrap *p*-values for the indices were obtained as twice the smaller of the proportions of resampled indices at or below and at or above zero. Confidence intervals are bias-corrected and are not accelerated. Descriptive comparisons between two groups used the Welch unequal-variance *t* test with the Mann–Whitney U test as a rank-based confirmation, comparisons across the three trimesters used one-way analysis of variance with Kruskal–Wallis confirmation, and comparisons of proportions used the chi-squared test; these tests are descriptive and are reported without correction for multiplicity. Analyses were conducted using Python version 3.11 (Python Software Foundation, Wilmington, DE, USA), with NumPy version 2.4, SciPy version 1.17, and pandas version 3.0. A fixed random seed was used, and all analyses were independently reproduced from the raw data before resubmission; the analysis code is deposited as stated in the Data Availability Statement.

The analysis decomposed the observed association between each standardized thyroid exposure and the composite antenatal distress outcome into a direct component and indirect components running through DERS-16 difficulties and ERSQ skills, specified as two parallel mediators.

Indirect associations and their bias-corrected 95% confidence intervals (CIs) came from 5000 bootstrap resamples under a fixed random seed for reproducibility, and an association was considered statistically supported when its interval excluded zero. To control false-positive inference, we applied the Benjamini–Hochberg false-discovery-rate procedure [36] to the primary indirect-association estimates and, as a separate family, to the four route-specific moderation indices. We quantified the sensitivity of the primary total associations to unmeasured confounding with E-values, computed for the point estimates and for the confidence limits nearest the null [37]. E-values were obtained by converting the standardized association coefficients to approximate risk ratios using the relation RR ≈ exp(0.91 × d) and applying E = RR + √(RR × (RR − 1)) so that the reported values are reproducible from the corresponding model coefficients. E-values were not extended to the indirect associations, because the E-value formalism applies to total effects and identification of an indirect association requires the additional and stronger assumption of no unmeasured mediator–outcome confounding. In keeping with the cross-sectional design, we report associations throughout and use total association in place of total effect.

Moderation was assessed with the index of moderated mediation, estimated separately for the DERS-16 and ERSQ routes and for two moderators, gestational week and log anti-thyroid-peroxidase titer [38,39]. For gestational age, we computed a bootstrap Johnson–Neyman grid [40] across gestational weeks to locate the region in which the DERS-16 conditional indirect association remained supported and obtained a bootstrap 95% confidence interval for the transition point by finding the region-of-significance boundary in each resample. The four distress instruments were specified beforehand as indicators of a single antenatal distress factor, and the fit and reliability of this measurement model were checked to confirm that one standardized composite score was an adequate operationalization of the outcome. Prespecified sensitivity analyses re-ran the primary models after stratifying by levothyroxine use, after removing the single highest observed anti-thyroid-peroxidase value, and using free T3 as the exposure. In response to peer review we added the following: a formal exposure-by-levothyroxine interaction test for both exposures; bootstrap confidence intervals for every reported stratified and sensitivity estimate; re-estimation of the primary models separately in women with non-quantifiable (< 28 U/mL) and quantifiable (≥ 28 U/mL) anti-thyroid-peroxidase titers; recomputation of the antibody-route index of moderated mediation after substituting censored values at the limit of quantification, at half that limit and at the limit divided by the square root of two; recomputation of the same index with the autoimmune marker recoded as an ordered three-level variable (non-quantifiable, quantifiable but below the positivity threshold, and positive); and re-estimation of the primary models after excluding women with gestational diabetes, women with an unplanned pregnancy, and both groups jointly. Because several strata contained too few women to support a two-mediator model with the full covariate set, and because in treated women current biochemistry reflects treatment adequacy, not underlying disease severity—so that stratification on a post-treatment quantity would not yield an interpretable contrast—we did not fit separate mediation models within biochemical phenotype strata; biochemical phenotype was examined descriptively. Target sample size was fixed a priori to exceed common recommendations for two-mediator moderated-mediation models, and an a priori Monte Carlo simulation (5000 replications, standardized a and b paths of about 0.3, two parallel mediators, and alpha of 0.05) indicated power above 0.90 for the total indirect association; power for the moderated-mediation index was lower, so those results are read cautiously. We did not compute retrospective (“observed”) power, which for a fixed sample size is a monotone transformation of the *p*-value and therefore conveys no information beyond the reported confidence intervals and whose use to explain non-significant findings is a recognized inferential error [41,42]. We instead characterized the precision of the design directly, by reporting the half-widths of the bias-corrected bootstrap confidence intervals for the primary indirect associations and for each index of moderated mediation, and by deriving, from the standard error implied by the reported bootstrap interval for each index, both the smallest index of moderated mediation detectable with 80% power at a two-sided alpha of 0.05 and the sample size that an index of the observed magnitude would require to reach that power. The analytic dataset contained no missing values on the analyzed variables; this reflects the exclusion after enrollment of 11 women whose questionnaire set was incomplete or whose same-visit biochemistry could not be linked (Section 3.1), rather than an absence of item non-response in the recruited sample.

## 3. Results

### 3.1. Participant Flow

**Figure 1:**
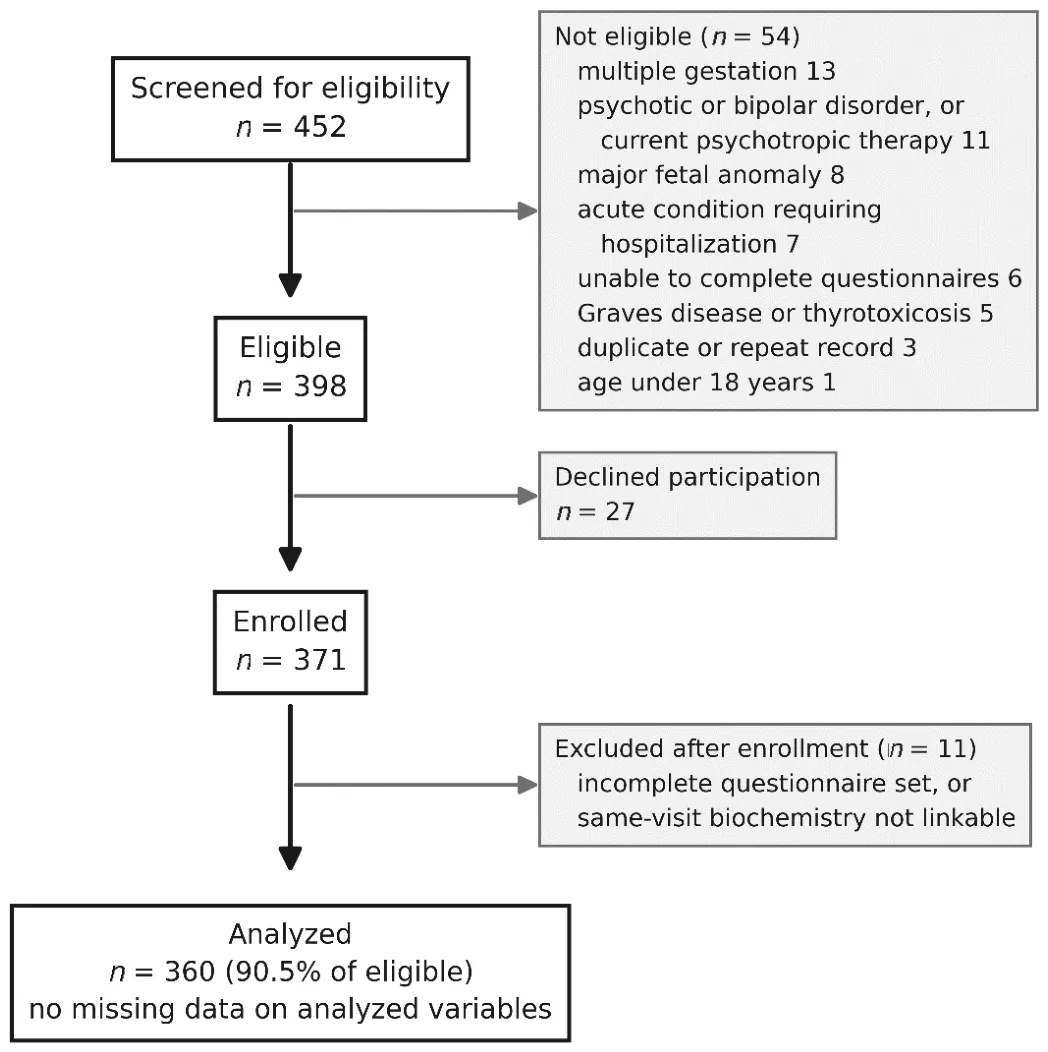
Participant flow. Numbers of women screened (*n* = 452), ineligible (*n* = 54, with the eight reasons itemized in the side box), eligible (*n* = 398), declining participation (*n* = 27), enrolled (*n* = 371), excluded after enrollment (*n* = 11, for an incomplete questionnaire set or unlinkable same-visit biochemistry), and analyzed (*n* = 360; 93.2% of eligible women participated and 90.5% of eligible women were analyzed, with no missing values on the analyzed variables). Diagram prepared according to the STROBE statement [16]. Black downward arrows indicate progression through the study cohort, whereas gray rightward arrows and gray-shaded boxes indicate exclusion or non-participation.

During the study period, 452 pregnant women with a documented diagnosis of hypothyroidism were screened at routine antenatal care. Fifty-four did not meet the eligibility criteria: multiple gestation (*n* = 13), a major fetal anomaly identified before assessment (*n* = 8), Graves disease or thyrotoxicosis (*n* = 5), current or past primary psychotic or bipolar disorder or current psychotropic pharmacotherapy (*n* = 11), an acute condition requiring hospitalization (*n* = 7), inability to complete the questionnaires because of literacy or language barriers (*n* = 6), age under 18 years (*n* = 1), and duplicate or repeat records (*n* = 3). Of the 398 eligible women, 27 declined, and a further 11 were excluded after enrollment because the questionnaire set was incomplete or the thyroid biochemistry from the same visit could not be linked. The final analytic sample comprised 360 women (90.5% of those eligible), with no missing values on the analyzed variables (Figure 1).

The participation rate among eligible women was therefore 93.2% (371/398), with 27 women (6.8%) declining to take part. Reasons for non-participation were not systematically recorded, so a reliable categorical breakdown cannot be provided, and no data were collected from women who declined; a formal comparison with participants was therefore not possible, and non-response bias cannot be excluded.

### 3.2. Sample Characteristics

Mean age was 29.4 years (standard deviation [SD] 5.0), and mean gestational age was 20.8 weeks (SD 9.3), spanning a wide range (6.6 to 38.8 weeks) that supported the test of gestational-age moderation. The trimester distribution was 109, 152, and 99 women in the first, second, and third trimesters. Hypothyroidism had been diagnosed before the index pregnancy in 174 women (48.3%), was first documented during the index pregnancy in 161 (44.7%), and could not be reliably classified in 25 (6.9%). Levothyroxine was used by 209 women (58.1%). At the manufacturer decision threshold of 60 U/mL, 97 women (26.9%) were anti-thyroid-peroxidase positive. A further 14 women (3.9%) had low-titer concentrations of 34 to 59 U/mL, above the limit of detection but below the decision threshold, and were classified as antibody-negative; the remaining 249 women (69.2%) had concentrations below 34 U/mL, of whom 243 (67.5% of the cohort) lay below the lower limit of the measuring interval. Median anti-thyroid-peroxidase concentration was 14.8 U/mL (interquartile range 9.9 to 72.0; range 2 to 1000). Mean TSH was 3.90 mIU/L (SD 2.79; median 3.27), mean free T4 was 0.959 ng/dL (SD 0.151), and mean free T3 was 2.76 pg/mL (SD 0.29). Composite antenatal distress did not differ across trimesters (mean standardized score −0.003, −0.004 and 0.010 in the first, second and third trimesters; one-way analysis of variance *p* = 0.99; Kruskal–Wallis *p* = 0.90), whereas free thyroxine (*p* = 0.010) and thyroid-stimulating hormone (*p* = 0.019) did. Table 1 presents the sample characteristics and scale scores. Table 2 summarizes the biochemical phenotype at the index antenatal visit. Table 3 presents the thyroid and psychological measures by trimester.

The cohort was clinically heterogeneous. Biochemical phenotype at the index visit is reported in Table 2 as a cross-classification of levothyroxine use, the thyroid-stimulating hormone band and the free thyroxine band, so that the cells partition the cohort exactly once instead of overlapping. No woman had a thyroid-stimulating hormone concentration below the non-pregnant lower limit of 0.55 mIU/L (observed minimum 0.67 mIU/L), so no over-replaced stratum was present. Among treated women, 130 (62.2% of those treated) had thyroid-stimulating hormone within the target and 79 (37.8%) above target. Among untreated women, 21 (13.9% of those untreated) met a subclinical and 35 (23.2%) an overt hypothyroid pattern, nine (6.0%) had isolated hypothyroxinemia, and 86 (57.0%) were biochemically euthyroid at the index visit on the basis of a documented historical diagnosis. That last group, 23.9% of the whole cohort, reflects the eligibility rule stated in Section 2.1: a documented diagnosis of hypothyroidism was sufficient for inclusion irrespective of the biochemistry obtained at the index visit.

Screening and biochemical thresholds were frequently met. A TSH of 4.0 mIU/L or higher occurred in 135 women (37.5%) and free T4 below 0.80 ng/dL in 51 (14.2%). Eighty-five women (23.6%) screened positive for probable depression at an EPDS total of 13 or above, 12 (3.3%) endorsed the EPDS self-harm item, and 66 (18.3%) reached the severe pregnancy-anxiety threshold; at least one threshold was met by 202 women (56.1%) (Table 4). These proportions describe screening status and referral burden, not diagnosed disorder. Internal consistency ranged from acceptable to excellent (DERS-16 alpha = 0.874; ERSQ alpha = 0.937; EPDS alpha = 0.753; PRAQ-R2 alpha = 0.806; CWS alpha = 0.875; PrAS alpha = 0.929). The DERS-16 and ERSQ totals were only weakly and non-significantly correlated (r = −0.052, *p* = 0.326). This is smaller in magnitude than the correlations typically reported between difficulties-based and skills-based emotion-regulation measures, and it is not attributable to unreliability, since disattenuating for the observed internal consistencies changes it only to −0.058. We verified the scoring direction of the Turkish ERSQ against the published scoring key and confirmed the expected inverse association with depressive symptoms (r = −0.25 with the EPDS, Table 5). The near-orthogonality of the two instruments in this sample is therefore consistent with their treatment as complementary rather than opposing dimensions, but it should be read as a sample-specific finding requiring replication, not as established discriminant validity. The single-factor model for antenatal distress fit well (χ^2^(2) = 0.58, CFI = 1.00, TLI = 1.00, RMSEA = 0.00, SRMR = 0.01), with standardized loadings from 0.58 to 0.89 and McDonald’s omega of 0.80. Because a four-indicator single-factor model has only two degrees of freedom and is nearly saturated, we read these indices as consistent with unidimensionality rather than as strong confirmatory evidence. Screening results and biochemical threshold summaries are presented in Table 4.

### 3.3. Bivariate Associations

**Figure 2:**
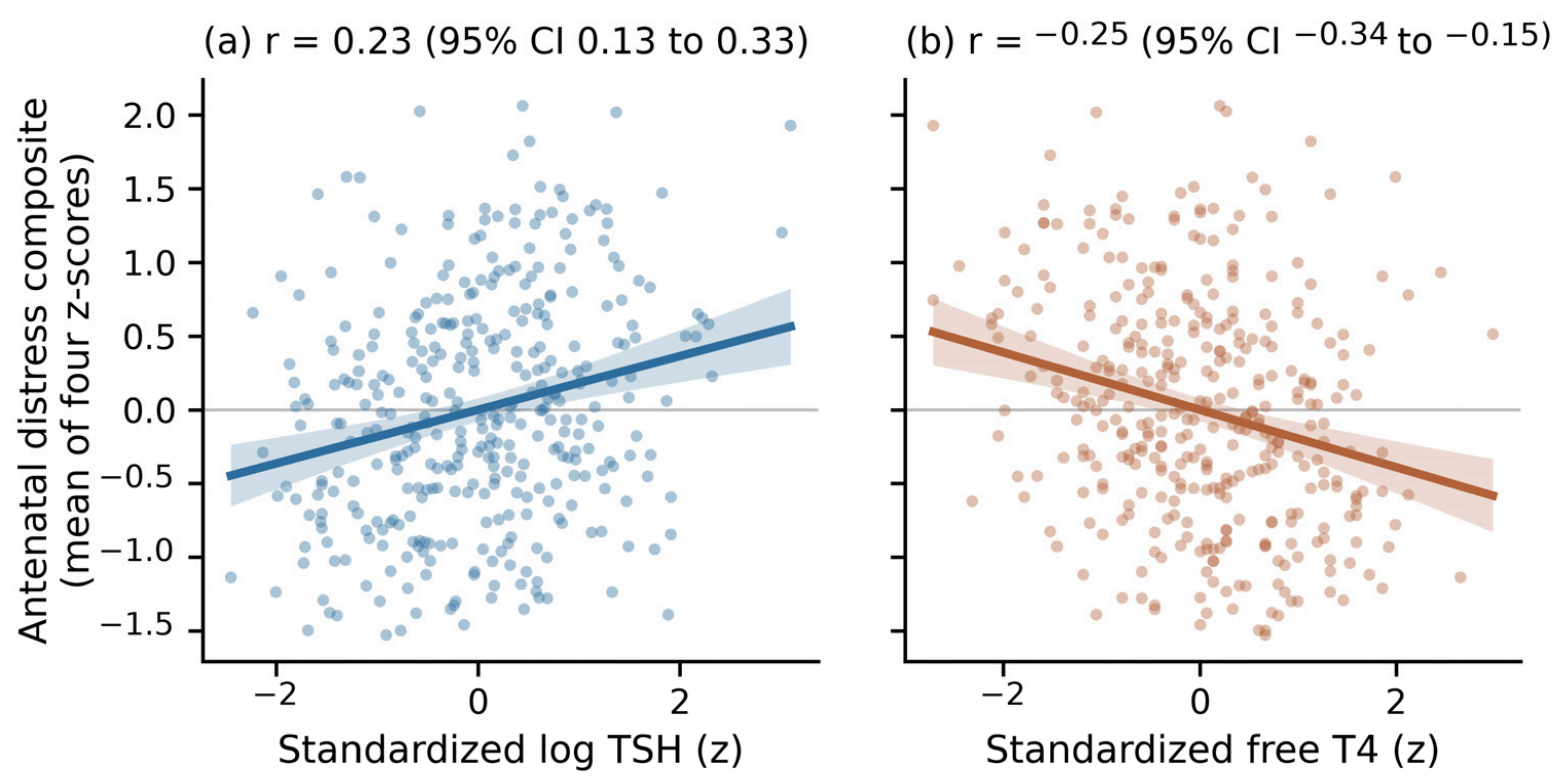
Thyroid biochemistry and antenatal distress (*n* = 360). Scatterplots with fitted regression lines and 95% confidence bands show the association of (**a**) standardized log TSH and (**b**) standardized free T4 with the composite antenatal distress score. The exposure axes are in standard-deviation (z) units. Points are drawn with partial transparency to show density. These fitted lines are unadjusted bivariate associations; all estimates reported in the text and in Table 6 are adjusted for the covariates listed in Section 2.4. Panel (**a**) *r* = 0.23 (95% CI 0.13 to 0.33); panel (**b**) *r* = −0.25 (95% CI −0.34 to −0.15). Dots represent individual participants, and the shaded areas indicate the 95% confidence bands around the fitted regression lines.

Bivariate patterns matched the hypothesized biological–psychological model. Higher log TSH and lower free T4 were each associated with greater emotion-regulation difficulties, poorer skills, and higher distress, while free T3 showed weaker associations in the same direction. Log anti-thyroid-peroxidase titer was modestly associated with higher distress (r of approximately 0.17). Among the psychological measures, DERS-16 difficulties correlated most strongly with composite distress (r = 0.40), and ERSQ skills correlated inversely (r = −0.30). Table 5 presents the correlations among the principal variables. Figure 2 shows the associations of standardized log TSH and free T4 with distress.

### 3.4. Primary Indirect-Association Models

Higher standardized log TSH was associated with greater distress: the total association was 0.189 (95% CI 0.108 to 0.272), and the total indirect association through DERS-16 difficulties and ERSQ skills was 0.141 (95% CI 0.093 to 0.192), split between a DERS-16 component of 0.101 (95% CI 0.060 to 0.144) and an ERSQ component of 0.040 (95% CI 0.018 to 0.069). The direct association was smaller and imprecise (0.048, 95% CI −0.028 to 0.128). Lower standardized free T4 produced the mirror pattern: a total association of −0.200 (95% CI −0.278 to −0.120), a total indirect association of −0.146 (95% CI −0.199 to −0.098), and a direct association whose interval crossed zero (−0.054, 95% CI −0.135 to 0.023). For both exposures, the indirect path accounted for most of the total association and the direct path was small (Table 6). The E-value for the total association was 1.66 for log TSH and 1.69 for free T4 and 1.44 and 1.47 for the respective confidence limits closest to the null. These values are modest. An unmeasured confounder would need to be associated with both the thyroid marker and antenatal distress by a risk ratio of approximately 1.7 each, above and beyond the measured covariates, to explain away the point estimates entirely and by approximately 1.45 each to move the confidence interval to include the null. Confounders of that strength are plausible in this setting: prior psychiatric history, which was unavailable in this dataset, is associated with antenatal distress considerably more strongly than this and would be sufficient on its own if it were also associated with thyroid biochemistry at comparable strength.

### 3.5. Moderation and Sensitivity Analyses

**Figure 3:**
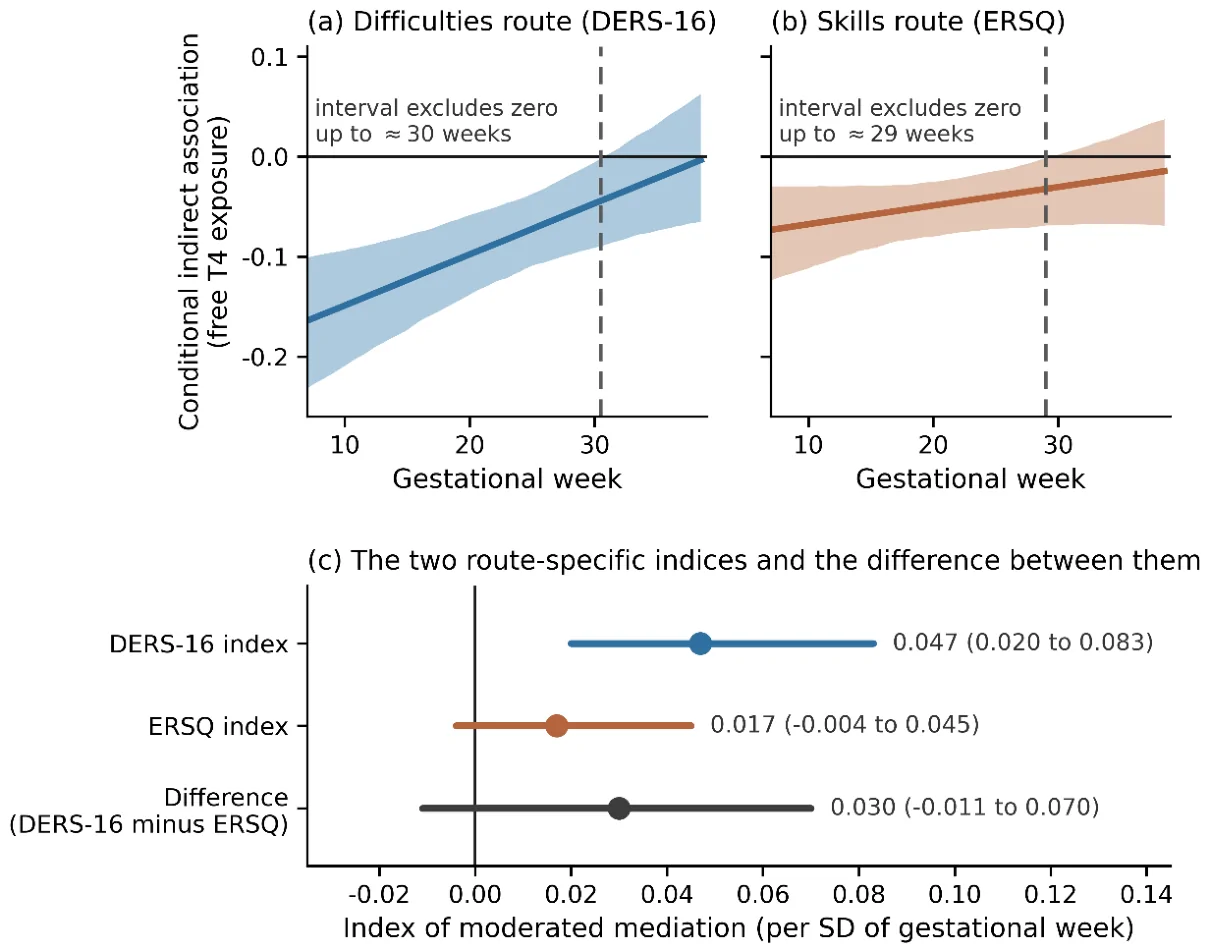
Conditional indirect associations across gestational week for the free T4 exposure, by mediating route, and the difference between the two route-specific indices. Panels (**a**,**b**) are plotted on a shared y-axis: the solid line is the point estimate and the shaded band the bias-corrected bootstrap 95% confidence interval; the horizontal reference line marks zero, and the dashed vertical line marks the gestational week beyond which the interval no longer excludes zero (approximately 30 weeks for the difficulties route and 29 weeks for the skills route). That boundary is estimated with limited precision and should not be read as a threshold. Panel (**c**) presents the two indices of moderated mediation and their difference (DERS-16 minus ERSQ), each with its bias-corrected bootstrap 95% confidence interval, plotted against a zero reference line: DERS-16 0.047 (0.020 to 0.083), ERSQ 0.017 (−0.004 to 0.045), and difference 0.030 (−0.011 to 0.070). The difference does not exclude zero, so the figure must not be read as showing that gestational age moderates the difficulties route specifically. Curves are not extrapolated beyond the observed range of gestational age (6.6 to 38.8 weeks).

The four route-specific indices of moderated mediation were evaluated together under Benjamini–Hochberg control. Gestational age moderated the difficulties (DERS-16) route, with an index that excluded zero (0.047, 95% CI 0.020 to 0.083; adjusted *p* = 0.002), whereas the skills (ERSQ) route was not moderated by gestational age (0.017, 95% CI −0.004 to 0.045; adjusted *p* = 0.18). In the bootstrap Johnson–Neyman grid, the difficulties-route conditional indirect association was strongest early in pregnancy and remained supported to approximately 30 weeks, with a bootstrap 95% confidence interval for the transition point running from about 26 to 38 weeks, so the boundary is estimated with limited precision (Figure 3). The skills-route (ERSQ) index for anti-thyroid-peroxidase positivity, defined at the manufacturer decision threshold of 60 U/mL, excluded zero (−0.027, 95% CI −0.053 to −0.008; adjusted *p* = 0.013), whereas the difficulties-route (DERS-16) index did not (−0.014, 95% CI −0.044 to 0.013; adjusted *p* = 0.38); both indices were negative, indicating that for the free T4 exposure the corresponding indirect associations became larger in magnitude in antibody-positive women. Because quantitative reporting below 28 U/mL is not validated and two-thirds of the results lie in that region, the binary marker, and not the continuous concentration, is the primary representation of thyroid autoimmunity in this cohort; the continuous coding is reported as a sensitivity analysis. Antibody-positive women had higher TSH than antibody-negative women (4.98 vs. 3.50 mIU/L, *p* = 0.0001), lower free T4 (0.907 vs. 0.979 ng/dL, *p* < 0.0001), higher composite distress (0.236 vs. −0.087, *p* = 0.0007), greater emotion-regulation difficulties (DERS-16 47.2 vs. 41.9, *p* = 0.0002) and poorer regulation skills (ERSQ 1.76 vs. 2.01, *p* = 0.0054); all five contrasts held in the same direction under the legacy 34 U/mL coding. For gestational week, the difference between the difficulties-route (0.047) and skills-route (0.017) indices was 0.030 (bias-corrected bootstrap 95% CI −0.011 to 0.070). For anti-thyroid-peroxidase positivity, both indices were negative, and the difference between the difficulties-route (−0.014) and skills-route (−0.027) indices was 0.013 (bias-corrected bootstrap 95% CI −0.024 to 0.047). Neither difference excluded zero (Table 7, difference rows). These data therefore do not establish that gestational age moderates the difficulties route specifically or that thyroid autoimmunity moderates the skills route specifically, and the route-specific pattern is reported as exploratory and hypothesis-generating throughout, including in Figure 3, which displays both routes and the difference between their indices.

Observing that one route-specific index excludes zero while the other does not is not a test of the difference between them. We therefore tested that difference directly, computing it within each of the same bootstrap resamples so that the covariance between the two indices is respected.

Sensitivity analyses supported the stability of the primary findings. Associations ran in the same direction in levothyroxine users and non-users, with confidence intervals now reported for every stratified estimate in Table 6. The formal exposure-by-treatment interaction was −0.004 (95% CI −0.140 to 0.141, *p* = 0.97) for the free T4 exposure and −0.063 (95% CI −0.211 to 0.075, *p* = 0.37) for log TSH, providing no evidence that the association differed by treatment status, although the confidence intervals do not exclude moderate differences and this analysis is not a substitute for the unavailable data on treatment duration and adherence. Dropping the single highest observed anti-thyroid-peroxidase value (1000 U/mL) left the estimates essentially unchanged (log TSH total 0.188, indirect 0.138; free T4 total −0.198, indirect −0.143). Free T3 models were weaker than the TSH and free T4 models but remained directionally concordant (Table 6).

Because the analytic file for the original submission contained a legacy binary antibody variable coded at a threshold of 34 U/mL rather than at the manufacturer decision threshold, and because two-thirds of results lie below the lower limit of the measuring interval, we examined the sensitivity of the antibody findings to the coding of this variable in four ways. First, the skills-route index of moderated mediation was recomputed with positivity defined at 34 U/mL (*n* = 111 positive, 30.8%): the index was −0.030 (95% CI −0.057 to −0.010), against −0.027 (95% CI −0.053 to −0.008) at the 60 U/mL threshold used for the primary analysis. Second, because a constant substituted for values below a reporting limit creates an artificial spike in the distribution and can distort correlation and regression estimates [43], the index was recomputed with the natural-log-transformed continuous concentration as the moderator under four handlings of the sub-interval results: as reported, −0.028 (95% CI −0.056 to −0.008); substituted at the lower limit of the measuring interval, 28 U/mL, −0.026 (95% CI −0.054 to −0.006); substituted at half that limit, 14 U/mL, −0.028 (95% CI −0.056 to −0.008); and substituted at the limit divided by the square root of two, 19.8 U/mL, −0.027 (95% CI −0.055 to −0.007). The corresponding difficulties-route indices lay between −0.013 and −0.017 and none excluded zero, and under no handling did the difference between the two route-specific indices exclude zero. Third, the moderator was recoded as an ordered three-level variable—below the measuring interval, quantifiable but below the decision threshold, and positive—yielding −0.029 (95% CI −0.056 to −0.009). Fourth, the primary indirect-association models were re-estimated separately in the 243 women whose anti-thyroid-peroxidase concentration lay below 28 U/mL and the 117 at or above it, for both exposures and for both routes. For free T4 the total indirect association was −0.100 (95% CI −0.161 to −0.049) below the limit and −0.174 (95% CI −0.302 to −0.089) at or above it, with difficulties-route components of −0.071 (95% CI −0.120 to −0.036) and −0.131 (95% CI −0.239 to −0.065) and skills-route components of −0.028 (95% CI −0.069 to 0.004) and −0.043 (95% CI −0.109 to 0.006). For log TSH the total indirect association was 0.118 (95% CI 0.067 to 0.181) and 0.150 (95% CI 0.068 to 0.275), with difficulties-route components of 0.078 (95% CI 0.039 to 0.132) and 0.128 (95% CI 0.057 to 0.249) and skills-route components of 0.040 (95% CI 0.009 to 0.080) and 0.021 (95% CI −0.001 to 0.086). Every component ran in the same direction in both strata. The indirect associations were larger in the stratum with quantifiable antibody concentrations, which is consistent with the antibody moderation reported above; because the lower stratum is by definition the one in which concentration is not quantitative, this contrast is a check on robustness, not a quantitative comparison of magnitude. The skills-route index excluded zero under every coding, and the difference between the two route-specific indices excluded zero under none of them, so neither the antibody finding nor the absence of route specificity depends on how the antibody variable is defined.

Gestational diabetes and unplanned pregnancy were adjusted for in all primary models. As a robustness check we re-estimated the primary models after excluding women with gestational diabetes (*n* = 15; total −0.197, 95% CI −0.275 to −0.115; indirect −0.147, 95% CI −0.196 to −0.098), women with an unplanned pregnancy (*n* = 47; total −0.207, 95% CI −0.293 to −0.121; indirect −0.126, 95% CI −0.176 to −0.080), and both groups jointly (analytic *n* = 298; total −0.204, 95% CI −0.290 to −0.118; indirect −0.127, 95% CI −0.176 to −0.080). All three restricted estimates were materially unchanged from the primary estimates of −0.200 and −0.146. Because gestational diabetes was recorded at the index antenatal visit, women screened after assessment could not yet be classified, so this restriction removes prevalent cases but not cumulative ones and is a conservative check, not a complete one.

### 3.6. Precision of the Design

The bias-corrected bootstrap confidence interval for the total indirect association had a half-width of 0.050 for log TSH and 0.051 for free T4, corresponding to a relative precision of approximately 35% of the point estimate for both exposures. Precision for the moderation indices was lower, with half-widths of 0.032 for the gestational-week difficulties-route index and 0.022 for the antibody skills-route index. Taking the reported interval for the skills-route index to imply a standard error of 0.011 on the standardized scale, the smallest index of moderated mediation that this design could detect with 80% power at a two-sided alpha of 0.05 is 0.032; an index of the magnitude actually observed on that route (0.027) would require approximately 510 participants to be detected with 80% power. The design was therefore well powered for the total indirect associations, which are estimated with narrow intervals, but only modestly powered for the moderation indices. This is a quantitative statement of the caution already applied to those results, and it gives a concrete sample-size target for the confirmatory study we recommend.

## 4. Discussion

Among these 360 pregnant women with hypothyroidism, higher TSH and lower free T4 were associated with greater antenatal distress, and two complementary emotion-regulation dimensions captured most of that association. Greater difficulties in emotion regulation and poorer adaptive skills each carried part of it, and together they accounted for the majority of the total association for both thyroid exposures. Once these dimensions were taken into account, the residual direct associations were small and their intervals included zero. Because every measurement came from one antenatal visit, these results show how the thyroid–distress association can be decomposed statistically; they do not show that thyroid dysfunction causes distress by impairing emotion regulation.

These estimates can be placed against the published literature. The direction we observe, higher thyroid-stimulating hormone and lower free thyroxine with greater distress, is the direction reported in the population-based synthesis linking hypothyroidism to clinical depression [1] and in the gestational thyroid-function literature [6]. The magnitude, a standardized total association of 0.189 for log thyroid-stimulating hormone and −0.200 for free thyroxine, is of the modest size those syntheses report, not the size that a strong biological determinant would produce. The rates in our own cohort are equally unremarkable: 23.6% screened positive on the Edinburgh Postnatal Depression Scale, within the 14.6% to 27.5% reported for Turkish antenatal populations [13,14], so the sample is neither unusually distressed nor unusually well. What the present analysis adds to this literature is therefore not the size of the thyroid–distress association, which is consistent with what is already known, but the finding that most of it is shared with two concurrently measured regulatory dimensions, a decomposition that the studies reporting the association alone [1,6,8] could not perform, because emotion regulation was not measured in them.

Two aspects of the pattern are clinically informative. The index of moderated mediation for the difficulties dimension excluded zero: the DERS-16 conditional indirect association was strongest early in pregnancy and lost support after about 31 weeks, though the wide bootstrap interval around that boundary means its position is approximate, and because the corresponding index for the skills route did not differ from it the pattern is descriptive, not route-specific. This fits the idea that difficulties in regulating emotion are most tightly coupled to thyroid-related distress during the period of greatest endocrine change and that early gestation is a more informative window for assessment. Thyroid autoimmunity was associated with the skills-route indirect association reaching statistical support, although the difference between routes was not significant: the antibody index for that route excluded zero and survived correction across the moderation family, and the direction was consistent under every antibody coding we examined (Section 3.5). Antibody-positive women also had higher TSH and lower free T4, consistent with the dose-dependent antibody–thyroid-function relationship established in individual-participant-data meta-analyses [7,44], and higher distress, in line with the autoimmune literature [6]. The apparent separation of the two moderators onto different routes should be read with caution. It rests on one route-specific index reaching support while the other did not, and that contrast alone does not establish a difference between them. When we tested the difference directly, neither difference excluded zero (0.030, 95% CI −0.011 to 0.070 for gestational week; 0.013, 95% CI −0.024 to 0.047 for antibody positivity), and for anti-thyroid-peroxidase positivity both indices were negative and differed only in magnitude. We therefore present the route specificity as exploratory and hypothesis-generating, to be confirmed in longitudinal work with more power for interaction terms.

These results sit within a small but growing collection of literature on emotion regulation in pregnancy. Penner and Rutherford have argued that emotion regulation is an under-researched yet modifiable target for perinatal mental-health screening and intervention and have called specifically for studies that measure regulatory processes alongside established risk markers instead of inferring them [12]; the present analysis is one response to that call, in that it measures two complementary regulatory dimensions in the same women in whom the biological marker was assayed. A systematic review of the perinatal emotion-regulation literature has since found that engagement-oriented strategies act as protective factors and that avoidance and rumination act as risk factors for perinatal depression and anxiety, while noting how thin the quantitative evidence base remains [45]. In a Swedish national cohort, DERS-16 scores measured in the second trimester predicted elevated depressive symptoms from pregnancy through the postpartum period across seven assessments [46]—a design with the temporal ordering the present study lacks, and one that makes the regulatory dimension a plausible antecedent rather than only a correlate. The modern statement of the process model of emotion regulation [47] provides the framework within which difficulties and skills are treated as related but non-redundant. Our observation that the two dimensions were essentially uncorrelated in this sample, yet each carried part of the association with distress, is consistent with that framework and argues against collapsing them onto a single scale, although a correlation this close to zero is smaller than the inverse association usually reported between difficulty and skill measures and warrants replication.

Keeping TSH and free T4 as separate primary exposures is a methodological strength. A single thyroid adequacy index can assign the same value to women in clinically different states, for instance an over-treated woman with low TSH and high free T4 and an under-treated woman with high TSH and low free T4. Reporting the two markers separately preserves this information, and both yielded mirror-image indirect associations, which strengthens confidence in the direction of the findings. Free T3, included as a sensitivity exposure, pointed the same way, addressing the concern that the most biologically active hormone had been left out; its weaker signal is consistent with the narrower dynamic range of free T3 in this cohort.

Two features of the cohort’s biochemistry require comment, because both would otherwise be read as implausible. Mean free thyroxine (0.959 ng/dL) lay only slightly above the assay’s non-pregnant lower reference limit of 0.89 ng/dL, and a substantial proportion of values fell below it. This should not be read as an unusually high prevalence of hypothyroxinemia. Free thyroxine measured by competitive immunoassay declines progressively through gestation as thyroxine-binding globulin rises and serum albumin falls, and this decline is method-dependent and most pronounced in the second and third trimesters—precisely the period in which most participants were assessed, at a mean gestational age of 20.8 weeks [48,49]. Applying a non-pregnant lower reference limit to such a cohort will classify a large fraction of physiologically normal women as low. This is a recognized limitation of free thyroxine immunoassay in pregnancy, and it is the principal reason we analyzed free thyroxine as a continuous standardized marker rather than as a dichotomous diagnostic category, reserving the descriptive threshold of 0.80 ng/dL for clinical description only.

The proportion of women with detectable thyroid autoimmunity deserves comment, because at 26.9% it is lower than would be expected if the cohort were composed predominantly of chronic autoimmune thyroiditis, which is the leading cause of hypothyroidism in iodine-sufficient populations [2,18]. Three features of this cohort account for the difference. First, eligibility rested on a documented historical diagnosis of thyroid dysfunction and not on biochemical or serological confirmation at the index visit, so the cohort includes women whose original diagnosis may have followed transient, postpartum, iodine-related or otherwise non-autoimmune dysfunction; 86 women (23.9%) were untreated and biochemically euthyroid when studied. Second, and most important, the diagnosis was first documented during the index pregnancy in 161 women (44.7%). Gestational-onset thyroid dysfunction frequently reflects the increased thyroidal demand of pregnancy and not autoimmune destruction, so a cohort in which nearly half the diagnoses arose in pregnancy will contain a correspondingly smaller autoimmune fraction. Third, long-term levothyroxine treatment is associated with a progressive fall in anti-thyroid-peroxidase titer [50], and 209 women (58.1%) were receiving replacement. Gestational decline in antibody titer does not explain the finding here: positivity did not differ across trimesters (25.7%, 31.6% and 21.2%; *p* = 0.18). Our antibody variable should therefore be read as detectable thyroid autoimmunity at the index antenatal visit rather than as the proportion of this cohort whose thyroid dysfunction is autoimmune in origin, and the moderation finding should be understood in those terms.

A second measurement point concerns the definition of antibody positivity. Antibody cut-offs are method-specific and are not interchangeable between platforms. In preparing this revision we found that the binary antibody variable in our original analytic file had been constructed at a threshold of 34 U/mL during research-dataset preparation rather than at the manufacturer decision threshold of 60 U/mL stated in our Materials and Methods. The laboratory has confirmed that no threshold of 34 or 35 U/mL was ever configured for patient reporting, that no locally derived decision threshold was in use, and that no migration from another immunoassay platform occurred during the study period. All analyses have been recomputed at 60 U/mL, which reclassified 14 women (3.9%) with concentrations of 34 to 59 U/mL from positive to negative. The direction, magnitude and statistical support of every antibody-related result were unchanged, and the skills-route index was marginally stronger at the manufacturer threshold, so our conclusions do not depend on the threshold applied.

Anti-thyroid-peroxidase antibodies were positive in a lower proportion of this cohort than would be expected if chronic autoimmune thyroiditis accounted for the great majority of hypothyroidism in this population. Several factors plausibly contribute: the manufacturer’s positivity threshold is relatively high and will classify some genuinely autoimmune women as negative; seronegative autoimmune thyroiditis cannot be identified without ultrasonography, which was not routinely performed; non-autoimmune etiologies, including post-surgical and post-ablative hypothyroidism, are represented in the cohort; and eligibility rested on a documented diagnosis of hypothyroidism, so that women whose biochemistry had normalized remained eligible. Thyroid autoimmunity in this cohort should therefore be understood as identified with specificity but incomplete sensitivity, which would tend to attenuate rather than inflate the moderation by antibody status that we report.

Levothyroxine treatment warrants comment. More than half the cohort was treated, and in treated women current biochemistry reflects treatment adequacy, not untreated disease severity. Stratified analyses showed associations in the same direction in treated and untreated women; they were somewhat weaker among the treated, with no clear interaction. Stratification by treatment status distinguishes only whether a woman was prescribed levothyroxine and cannot capture treatment intensity, so current biochemistry in treated women reflects the adequacy of replacement rather than the severity of underlying disease. Residual confounding by treatment intensity therefore remains, and it would act in an unpredictable direction: under-replacement would tend to align higher thyroid-stimulating hormone with longer-standing untreated disease, whereas good adherence would tend to attenuate the exposure contrast. Because adherence is unknown, the translational claim that thyroid biochemistry can flag women for emotion-regulation assessment is correspondingly weaker in treated women, and we do not extend it to any inference about treatment adequacy. Because maternal thyroid status and thyroid autoimmunity also affect fetal neurodevelopment and neonatal thyroid function [51], identifying women whose distress is coupled to thyroid dysregulation matters beyond maternal wellbeing.

The literature on thyroid-stimulating hormone and antenatal mood is genuinely inconsistent, and our positive finding should be placed against that background [6,52]. Several sources of heterogeneity are apparent. First, thresholds differ: studies applying a fixed 2.5 mIU/L upper limit, a fixed 4.0 mIU/L limit, or locally derived trimester-specific intervals will classify markedly different proportions of the same population as abnormal, and dichotomized analyses are correspondingly sensitive to that choice. In Turkish antenatal cohorts this choice alone moves the reported prevalence of gestational hypothyroidism from 22.3% under a fixed 2.5 mIU/L limit to 2.9% under a fixed 4.0 mIU/L limit [15]. That is an inconsistency of definition, not of biology, as anticipated in the Introduction. Second, source populations differ: cohorts drawn from unselected pregnant women, in whom thyroid-stimulating hormone varies over a narrow range, have limited exposure contrast, whereas cohorts such as ours, restricted to women with diagnosed hypothyroidism, span a wider range and therefore have greater power to detect a graded association. Third, gestational timing differs: thyroid-stimulating hormone is physiologically suppressed by human chorionic gonadotrophin in the first trimester and rises thereafter, so cross-sectional samples drawn at different gestations are not directly comparable—an effect our own gestational-age moderation analysis makes visible and one that means the early-gestation signal we report arises in a period of considerable physiological variation in the exposure itself. Fourth, treatment status differs: in treated women, thyroid-stimulating hormone reflects treatment adequacy, not disease severity, so cohorts with differing proportions of treated women are estimating subtly different quantities. Fifth, outcome measurement differs: studies using a single depression screen, a diagnostic interview, or a multi-instrument composite such as ours capture different bandwidths of distress, and the wider bandwidth of a composite will generally yield a stronger association with a continuous exposure. Our analysis addressed the first, third and fourth of these directly—by retaining thyroid-stimulating hormone as a continuous standardized exposure rather than dichotomizing it, by modeling gestational age as a moderator, and by stratifying on levothyroxine use—and we suggest that the apparent inconsistency in this literature reflects these design differences at least as much as any true heterogeneity of effect.

### 4.1. Clinical Implications

These findings identify a correlate, not an intervention target. Because temporal ordering is not identified, we cannot infer that improving emotion regulation would reduce distress in women with hypothyroidism, nor that treating thyroid dysfunction would improve emotion regulation; the relevant trials in adjacent questions are a caution here, since levothyroxine treatment of subclinical hypothyroidism and hypothyroxinemia in pregnancy has not reliably improved the outcomes it was expected to improve [53,54], so biochemical correction cannot be assumed to be outcome correction. What the results do support is a risk-stratification observation: in this cohort, women with higher thyroid-stimulating hormone, lower free thyroxine or thyroid autoimmunity also had greater emotion-regulation difficulties and poorer regulation skills, and those dimensions accounted for most of the observed association with distress. That co-occurrence is a reason to consider co-screening within the antenatal pathway that already exists for these women, and it can be specified concretely. Women attending for routine antenatal review of hypothyroidism, and particularly those with higher thyroid-stimulating hormone, lower free thyroxine, thyroid autoimmunity, or assessment early in gestation, could complete a brief emotion-regulation measure—the 16-item DERS-16 takes approximately five minutes—at the same visit at which thyroid biochemistry is drawn and the EPDS is administered, most usefully at the booking or first-trimester review, when the association we observed was strongest. A positive depression screen at the conventional EPDS threshold of 13 or above, or endorsement of the self-harm item, already triggers referral to perinatal mental-health services under existing protocols; the proposal here is that an accompanying elevation on the difficulties dimension be used to prioritize referral and to indicate the content of that referral, since brief skills-focused psychoeducational and cognitive-behavioral interventions target precisely the capacities the skills dimension names. Because the EPDS and the pregnancy-anxiety instruments are screening tools, a positive result on any of them indicates a need for diagnostic assessment, not a diagnosis, and the emotion-regulation measures proposed here are likewise descriptive, not diagnostic. Establishing that this co-screening step improves outcomes would require a prospective study in which emotion regulation is measured before the outcome and, ultimately, a randomized comparison of the augmented pathway against usual antenatal screening—a design we are pursuing and which the present cross-sectional analysis motivates but does not replace. The difficulties dimension is well suited to flagging regulation problems amenable to brief, skills-focused intervention, psychoeducation, and cognitive-behavioral strategies, with referral to perinatal mental-health care when safety thresholds are met; the skills dimension names the capacities such interventions aim to build. Understood in these terms, the study contributes to risk stratification and care coordination, not to a claim that thyroid dysfunction causally produces distress through emotion regulation.

A practical qualification applies to any screening recommendation arising from these data. Because adherence to levothyroxine was unknown, an elevated thyroid-stimulating hormone concentration at a single visit does not distinguish inadequate prescribing from inadequate absorption or inadequate adherence, and a distress-related mechanism cannot be separated from a behavioral one. The clinical inference we draw is therefore limited to case identification—that women with less favorable thyroid biochemistry warrant closer attention to emotional wellbeing—and does not extend to any claim that optimizing thyroid biochemistry will reduce distress.

### 4.2. Strengths and Limitations

The study has real strengths. The sample is adequate for a two-mediator moderated-mediation analysis, the thyroid phenotype is detailed, and the psychological phenotype is broad and internally consistent. Reporting the Johnson–Neyman boundary with a bootstrap interval and controlling the false discovery rate across the moderation family address recurrent methodological concerns in this literature, and E-values put a number on how vulnerable the primary associations are to unmeasured confounding without demonstrating that they withstand it. As requested in review we report the precision of the design and the smallest index of moderated mediation it could have detected (Section 3.6) in place of retrospective power, which at a fixed sample size is a monotone transformation of the *p*-value and adds nothing to the reported confidence intervals [41,42]. One design feature deserves emphasis because it removes a specific alternative explanation: all questionnaires were completed before any thyroid result from the index visit was available to or communicated with the participant. Knowledge of an abnormal thyroid-stimulating hormone value could itself raise anxiety and generate a spurious thyroid–distress association; that channel is closed by the administration protocol, so the observed covariance cannot be attributed to result disclosure at the index visit.

The design has some limitations. The design is cross-sectional and rests on a single antenatal blood draw and questionnaire, whereas thyroid physiology and emotional symptoms both change across pregnancy. Because exposure, mediators and outcome were measured on one occasion, their temporal ordering is not identified: the regulatory dimensions could stand in a reciprocal rather than a one-directional relationship with distress, and a reverse ordering in which persistent distress affects both regulatory capacity and thyroid biochemistry is equally compatible with the observed covariance structure. Estimates of indirect effects obtained from cross-sectional data are known to be biased in a direction and magnitude that cannot be determined without longitudinal data [55]. We therefore report a decomposition of association throughout and make no causal claim. The design that would test the pathway properly is a prospective cohort with repeated thyroid and psychological measurement in which the regulatory dimensions are measured between the exposure and the outcome assessments, and we regard the present findings as motivating such a study, not substituting for one. There were also measurement limitations. Several limitations follow from the single-occasion, self-report design. No structured or clinical diagnostic interview was administered, so this study reports screening and symptom-dimension scores and cannot estimate the prevalence of diagnosed depressive or anxiety disorders; the screen-positive proportions in Table 4 describe referral burden, not case status. All psychological constructs were assessed on one occasion, so only internal-consistency reliability could be estimated; test–retest reliability and within-woman stability are unknown in this cohort. The four distress instruments index current symptom states over short reference windows, whereas the two emotion-regulation instruments index comparatively trait-like regulatory tendencies, so that the exposure, being a same-visit blood measurement, is matched in temporal resolution to the state outcome but not to the more trait-like mediators. The Turkish adaptation of the EPDS was validated in a postpartum sample, not an antenatal one, so the threshold of 13 used for descriptive reporting and safety referral is inherited from a different perinatal period; because the primary analyses used the continuous score as one of four indicators of a single distress dimension, this affects how the reported screen-positive proportion should be read, not the association estimates. Current levothyroxine dose was retrievable for 168 of the 209 treated women (80.4%) and is reported descriptively but was not modeled for the reasons given in Section 2.4; treatment duration, the history of dose adjustment and adherence could not be established for any participant, so biochemistry in treated women may reflect treatment adequacy and not disease severity. Prior psychiatric history, a strong predictor of antenatal distress, was unavailable, and post-referral outcomes for women meeting safety thresholds were not captured systematically, leaving residual confounding possible. Although all thyroid tests used one chemiluminescent platform in the same central laboratory, laboratory-derived trimester-specific pregnancy reference intervals were unavailable for these assays. In accordance with pregnancy-specific guidance [2,18] we therefore applied an upper thyroid-stimulating hormone reference limit of approximately 4.0 mIU/L, which for this assay is marginally more conservative than the guideline-suggested approach of reducing the non-pregnant upper limit of 4.78 mIU/L by approximately 0.5 mIU/L. Because that approximate limit derives predominantly from first-trimester data, whereas thyroid-stimulating hormone returns towards non-pregnant concentrations later in gestation, a uniform threshold applied across a cohort spanning 6.6 to 38.8 weeks will tend to over-classify women assessed later in pregnancy; the free thyroxine reference limits are likewise non-pregnant and, for the reasons set out in the Discussion, systematically under-classify free thyroxine in the second and third trimesters. These constraints affect only the descriptive threshold counts in Table 4, since all inferential analyses used continuous standardized markers. Routine thyroid ultrasonography was not performed. Although ultrasonography is not required to diagnose hypothyroidism, it can sometimes identify the diffusely hypoechoic, heterogeneous appearance characteristic of chronic autoimmune thyroiditis in women whose thyroid antibodies are negative, although its sensitivity in seronegative disease is limited [56]; because a majority of this cohort was anti-thyroid-peroxidase negative, some seronegative autoimmune disease will have been unclassified, and etiological subgrouping of the cohort is correspondingly incomplete. There are also inferential limitations. These constraints reinforce reading the results as a decomposition of association rather than causal mediation. The path-model outcome was a standardized composite of the four distress measures rather than a latent variable within a full structural equation model; the confirmatory factor model supported treating the four instruments as one dimension, but a composite does not propagate measurement error as a latent-variable model would, and such a model is a reasonable next step for confirmatory work. Two further points bear on interpretation. First, mediator and outcome were measured at the same visit, so their temporal order is not identified, and the regulatory dimensions could stand in a reciprocal rather than one-directional relationship with distress. Second, difficulties in emotion regulation and antenatal distress share negative-affect content, and the moderate correlation between the DERS-16 and the distress composite (r = 0.40) means the indirect association may partly reflect construct overlap rather than a wholly distinct regulatory process; the E-values bear only on the total associations, whereas the indirect estimates rest on the stronger assumption of no unmeasured mediator–outcome confounding, for which prior psychiatric history is a plausible candidate. Finally, two-thirds of anti-thyroid-peroxidase results lay below the lower limit of the assay measuring interval, below which quantitative reporting of patient results is not validated; 58.9% lay below the limit of detection and 42.5% below the limit of blank. Those results were analyzed as reported and were not replaced by a constant, so the continuous variable carries no artificial point mass, but it is not a quantitative measurement across most of its range; the continuous autoimmune analyses should therefore be taken as supporting the antibody-positivity results rather than as independent quantitative estimates. The well-characterized range between the reporting limit and the positivity threshold is narrow, so these analyses are underpowered to characterize precisely the low-titer range in which recent individual-participant-data work suggests clinically relevant information about maternal thyroid function resides and in which dichotomous and continuous codings can therefore diverge [44]. This is a limitation of the assay, not of the antibody. Anti-thyroglobulin antibody was measured but is not reported as a marker of thyroid autoimmunity and was not entered into any model. At the manufacturer threshold of 4.5 IU/mL, 359 of 360 women (99.7%) would have been classified as positive and the lowest observed concentration was 3.6 IU/mL, so no participant fell within the healthy reference range of below 1.3 IU/mL stated in the package insert. That is not consistent with the frequency of anti-thyroglobulin positivity reported in autoimmune hypothyroidism, and it is not reconcilable with the anti-thyroid-peroxidase results obtained from the same specimens, which would imply isolated anti-thyroglobulin positivity in approximately two-thirds of the cohort. The method identity and unit mapping of this variable could not be reconciled retrospectively with the expected Atellica IM aTgII reporting characteristics, and anti-thyroglobulin results were therefore excluded from all inferential analyses and from clinical interpretation instead of being reported as a positivity proportion we cannot substantiate.

At 1.66 and 1.69 for the total associations and 1.44 and 1.47 at the confidence limits closest to the null, the E-values are modest by the standards of this literature: a confounder associated with both the thyroid marker and distress at about a 1.7-fold ratio, over and above the measured covariates, would suffice to explain the point estimates away. The E-values are therefore best read as indicating that the observed total associations are not robust to moderate unmeasured confounding, and not as proof of robustness. This is a limitation of the design, not of the estimates.

A further measurement consideration is common-method variance. Although the exposure was a laboratory measurement and therefore shares no method with any psychological variable, both mediators and all four distress indicators were self-report questionnaires completed by the same respondent in a single sitting, so covariance among them may partly reflect the shared measurement method—response style, transient mood, item-context effects, or a consistency motif—rather than only the intended constructs [57]. Because the indirect association is the product of an exposure–mediator path and a mediator–outcome path, and only the latter is method-shared, upward bias in the mediator–outcome path would inflate the indirect estimate and correspondingly deflate the direct one. Three considerations bound this concern. First, the pattern of correlations is discriminant, not uniformly elevated: the DERS-16 and ERSQ totals were essentially uncorrelated (r = −0.052, *p* = 0.326), which is not what a pervasive method factor across a single-sitting self-report battery would produce, and the associations of the emotion-regulation measures with the four distress indicators ranged widely (0.23 to 0.44 for the DERS-16 and −0.19 to −0.31 for the ERSQ), whereas a dominant method factor produces a flat, uniformly inflated matrix. Second, the two scales are oppositely valenced, so a valence-driven response style would push their observed correlation negative; the observed value is trivially small on either reading. Third, common-method variance is not expected to generate spurious interaction effects and tends instead to attenuate them, so the moderated-mediation indices reported here are, if anything, conservative [58]. Nevertheless, because construct overlap between emotion dysregulation and negative affect and shared method variance cannot be fully separated in a single-occasion self-report design, the indirect estimates should be read as upper bounds on the regulatory contribution rather than as unbiased quantifications of it.

Generalizability is another limitation. Recruitment took place in a single tertiary perinatology unit, and participation among eligible women was high at 93.2%, which limits the scope for selection by willingness to disclose emotional difficulty. Three features nonetheless bound the population to which these findings apply. Women unable to complete written Turkish-language questionnaires were excluded at screening, so the findings may not extend to women with limited literacy or to non-Turkish-speaking women, nor to hypothyroid pregnancies managed in primary or secondary care. Women receiving psychotropic medication were excluded to avoid confounding of both thyroid function and emotional symptoms by pharmacological treatment; this necessarily removes women with recognized and treated depressive or anxiety disorders, which is part of the upper tail of the distress distribution, so the screen-positive proportion we report is a proportion among pharmacologically untreated women, and truncation of the outcome distribution in this way would be expected to attenuate rather than exaggerate the associations reported here. We also deliberately did not exclude women with coexisting conditions associated with antenatal distress: women with hypothyroidism in a tertiary perinatology setting frequently have additional obstetric or medical comorbidity, and excluding them would have produced a cohort unrepresentative of the population to whom any screening recommendation would apply—by the same logic we would have had to exclude women with low income (27.5%), limited social support (14.2%) and nulliparity (56.7%). Separately, the cohort was clinically heterogeneous, comprising women with treated hypothyroidism at differing degrees of biochemical control, with untreated subclinical and overt disease, and whose diagnosis was established but whose biochemistry had normalized (Table 2); the exposure contrast we report is therefore one of current biochemical status across a mixed population and not of disease severity within a uniformly defined condition, and our findings cannot be attributed to any single hypothyroid phenotype. The frequency of thyroid-function monitoring and of specialist review during gestation was not standardized for research purposes, and the number of antenatal contacts may itself relate to antenatal distress in either direction, through reassurance on the one hand or heightened illness salience on the other. Participants were also necessarily aware of their hypothyroidism, and more than half were under ongoing biochemical surveillance, so part of the observed association between biochemistry and distress may run through awareness of the biochemical result rather than through the biological state alone; the dataset did not record what each participant knew about her thyroid results at the time of completion, so this alternative, information-mediated pathway cannot be distinguished from a biological one. Finally, iodine status was not assessed, and mild iodine insufficiency continues to be reported among pregnant women in Türkiye [59]; neither urinary iodine concentration nor iodine-containing supplement use was recorded, so we cannot determine whether variation in iodine intake contributed to the biochemical variation observed.

## 5. Conclusions

In this cross-sectional study of hypothyroid pregnancy, higher thyroid-stimulating hormone and lower free thyroxine were associated with greater antenatal distress at the study visit, and two concurrently measured emotion-regulation dimensions statistically accounted for most of that association. The conditional indirect association through emotion-regulation difficulties was larger earlier in gestation, and the antibody-titer index for the skills route excluded zero; however, the difference between the two route-specific indices did not exclude zero for either moderator, so these data do not establish that either moderator acts on one route specifically, and both moderation findings are exploratory. Because exposures, mediators and outcomes were all measured at a single antenatal visit, temporal ordering is not identified and reverse or reciprocal orderings cannot be excluded; the findings are a decomposition of a cross-sectional association and not evidence of causal mediation, and the modest E-values indicate that they are not robust to moderate unmeasured confounding. Within these constraints, the results suggest that structured assessment of emotion regulation may be informative alongside established thyroid monitoring and depression screening in this group, and they motivate a longitudinal study in which the regulatory dimensions are measured between exposure and outcome.

## Figures and Tables

**Table 1:** Sample characteristics and scale scores (*N* = 360).

Characteristic	Value
Age, years, mean (SD)	29.4 (5.0)
Gestational age, weeks, mean (SD)	20.8 (9.3)
Trimester 1/2/3, *n*	109/152/99
Pre-pregnancy BMI, kg/m^2^, mean (SD)	25.6 (4.4)
Nulliparous, *n* (%)	204 (56.7)
University education, *n* (%)	133 (36.9)
Low income, *n* (%)	99 (27.5)
Low social support, *n* (%)	51 (14.2)
Current smoking, *n* (%)	37 (10.3)
Planned pregnancy, *n* (%)	313 (86.9)
Gestational diabetes, *n* (%)	15 (4.2)
Chronic hypertension, *n* (%)	4 (1.1)
Levothyroxine use, *n* (%)	209 (58.1)
TSH, mIU/L, mean (SD); median	3.90 (2.79); 3.27
Free T4, ng/dL, mean (SD)	0.959 (0.151)
Free T3, pg/mL, mean (SD)	2.76 (0.29)
Anti-TPO < 28 U/mL (below the measuring interval), *n* (%)	243 (67.5)
Anti-TPO 28–33 U/mL, *n* (%)	6 (1.7)
Anti-TPO 34–59 U/mL (low titer, below the decision threshold), *n* (%)	14 (3.9)
Anti-TPO ≥ 60 U/mL (positive; manufacturer decision threshold), *n* (%)	97 (26.9)
Anti-thyroglobulin, IU/mL, median (IQR)—not used in analyses	22.8 (15.1–53.4)
Anti-TPO, U/mL, median (IQR), whole sample	14.8 (9.9–72.0)
Anti-TPO ≥ 34 U/mL (legacy coding; sensitivity analyses only), *n* (%)	111 (30.8)
DERS-16 total, mean (SD)	43.3 (11.7)
ERSQ total, mean (SD)	1.95 (0.78)
EPDS total, mean (SD)	8.9 (5.6)
PRAQ-R2 total, mean (SD)	27.3 (8.1)
PrAS total, mean (SD)	75.4 (19.3)
CWS total, mean (SD)	31.3 (14.6)

BMI, body mass index; SD, standard deviation; TSH, thyroid-stimulating hormone; anti-TPO, anti-thyroid-peroxidase antibody; DERS-16, 16-item Difficulties in Emotion Regulation Scale; ERSQ, Emotion Regulation Skills Questionnaire; EPDS, Edinburgh Postnatal Depression Scale; PRAQ-R2, revised Pregnancy-Related Anxiety Questionnaire; PrAS, Pregnancy-related Anxiety Scale; CWS, Cambridge Worry Scale; IQR, interquartile range. Anti-thyroid-peroxidase concentrations are reported in U/mL and anti-thyroglobulin concentrations in IU/mL, as specified by the respective package inserts. The four anti-thyroid-peroxidase concentration bands are mutually exclusive and exhaustive and sum to *N* = 360. The binary autoimmune marker used in all primary models was defined at the manufacturer decision threshold of 60 U/mL; the row reporting 111 women at 34 U/mL corresponds to the legacy coding present in the original analytic file and is shown because it is used in the sensitivity analyses reported in Section 3.5. Quantitative reporting of patient results below 28 U/mL is not validated by the laboratory, so summary statistics for anti-thyroid-peroxidase should be read accordingly. Anti-thyroglobulin positivity is not tabulated: at the manufacturer threshold of 4.5 IU/mL, 359 of 360 specimens (99.7%) would be classified as positive, which is not reconcilable with the anti-thyroid-peroxidase results from the same specimens; the method identity and unit mapping of that variable could not be verified, and it is excluded from all analyses (Section 4.2).

**Table 2:** Biochemical phenotype at the index antenatal visit (*N* = 360).

Levothyroxine	TSH Band, mIU/L	Free T4 < 0.89 ng/dL, *n*	Free T4 ≥ 0.89 ng/dL, *n*	Total, *n* (%)	Descriptive Label
Yes	<0.55	0	0	0 (0.0)	Over-replacement
Yes	0.55–3.99	15	115	130 (36.1)	Treated, TSH within target
Yes	≥4.00	53	26	79 (21.9)	Treated, TSH above target
No	<0.55	0	0	0 (0.0)	Untreated, low TSH
No	0.55–3.99	9	86	95 (26.4)	Untreated, TSH in range
No	≥4.00	35	21	56 (15.6)	Untreated, TSH elevated
All	—	112	248	360 (100.0)	—

The three thyroid-stimulating hormone bands are disjoint, the two free thyroxine columns partition each band, and the two treatment strata are exhaustive; the twelve cells therefore tile the cohort exactly once and sum to the levothyroxine strata (209 and 151) and to *N* = 360. The descriptive labels in the final column are conventional shorthand for individual cells and are not additional categories: within the untreated, the thyroid-stimulating hormone ≥4.00 mIU/L band and women with free thyroxine below 0.89 ng/dL correspond to overt and those at or above 0.89 ng/dL to subclinical hypothyroidism, while untreated women with thyroid-stimulating hormone in range and free thyroxine below 0.89 ng/dL correspond to isolated hypothyroxinemia. Reference limits are the assay non-pregnant limits and are applied here for classification only; trimester-specific limits were unavailable for these assays (Section 4.2). These categories are descriptive and were not used to stratify the mediation models: several cells are too small to support a two-mediator model with the full covariate set, and in treated women current biochemistry reflects treatment adequacy, not underlying disease severity, so stratifying on a post-treatment quantity would not yield an interpretable contrast. TSH, thyroid-stimulating hormone.

**Table 3:** Thyroid and psychological measures by trimester (*N* = 360).

Measure	First Trimester (*n* = 109)	Second Trimester (*n* = 152)	Third Trimester (*n* = 99)	*p*
TSH, mIU/L, median (IQR)	3.14 (1.83–4.32)	3.09 (2.04–4.79)	3.84 (2.44–5.58)	0.019
TSH ≥ 4.0 mIU/L, *n* (%)	34 (31.2)	55 (36.2)	46 (46.5)	0.069
Free T4, ng/dL, mean (SD)	0.99 (0.15)	0.95 (0.16)	0.93 (0.13)	0.010
Free T3, pg/mL, mean (SD)	2.82 (0.27)	2.75 (0.30)	2.69 (0.28)	0.006
DERS-16 total, mean (SD)	41.08 (12.36)	44.08 (11.94)	44.59 (10.16)	0.054
ERSQ total, mean (SD)	1.91 (0.78)	1.87 (0.79)	2.10 (0.74)	0.067
EPDS total, mean (SD)	8.81 (6.06)	8.86 (5.51)	8.88 (5.31)	0.995
PRAQ-R2 total, mean (SD)	27.64 (8.21)	27.16 (7.95)	27.19 (8.17)	0.881
PrAS total, mean (SD)	76.65 (20.54)	74.30 (18.61)	75.65 (18.90)	0.617
CWS total, mean (SD)	29.69 (15.01)	32.13 (15.10)	31.86 (13.35)	0.376
Composite distress, mean (SD)	−0.00 (0.85)	−0.00 (0.77)	0.01 (0.74)	0.989

*p*-values are from one-way analysis of variance for continuous measures and the chi-squared test for proportions; the Kruskal–Wallis test gave concordant results. Trimesters are defined as <14, 14 to <28, and ≥28 completed weeks.

**Table 4:** Screening and biochemical threshold summary (*N* = 360).

Clinical Threshold	*n*	%
TSH ≥ 4.0 mIU/L	135	37.5
Free T4 < 0.80 ng/dL	51	14.2
EPDS ≥ 13	85	23.6
EPDS self-harm item ≥ 2	12	3.3
Severe pregnancy anxiety	66	18.3
Any clinical threshold met	202	56.1

EPDS and pregnancy-anxiety thresholds denote screen-positive status on self-report instruments and do not indicate diagnosed disorder; no diagnostic interview was administered. TSH and free T4 thresholds are descriptive, are applied uniformly across gestation, and derive predominantly from first-trimester data (see Section 4.2); all primary models used continuous standardized markers. TSH, thyroid-stimulating hormone; free T4, free thyroxine; EPDS, Edinburgh Postnatal Depression Scale.

**Table 5:** Correlations of thyroid and emotion-regulation measures with antenatal distress indicators.

Measure	Composite Distress	EPDS	PRAQ-R2	PrAS	CWS
Log TSH	0.23	0.21	0.17	0.22	0.13
Free T4	−0.25	−0.21	−0.20	−0.23	−0.15
Free T3	−0.18	−0.21	−0.15	−0.15	−0.05
DERS-16 difficulties	0.40	0.44	0.23	0.33	0.27
ERSQ skills	−0.30	−0.25	−0.20	−0.31	−0.19

Values are Pearson correlation coefficients. Composite distress is the standardized distress score. Log anti-thyroid-peroxidase titer was modestly associated with composite distress (*r* ≈ 0.17).

**Table 6:** Primary separate thyroid-exposure indirect-association models and sensitivity analyses.

Exposure	Path or Stratum	Estimate (95% CI)	Model
Log TSH (z)	Total association	0.189 (0.108 to 0.272)	Primary
Log TSH (z)	Total indirect	0.141 (0.093 to 0.192)	Primary
Log TSH (z)	Indirect via DERS-16	0.101 (0.060 to 0.144)	Primary
Log TSH (z)	Indirect via ERSQ	0.040 (0.018 to 0.069)	Primary
Log TSH (z)	Direct association	0.048 (−0.028 to 0.128)	Primary
Log TSH (z)	Total association, levothyroxine users (*n* = 209)	0.144 (0.019 to 0.256)	Sensitivity
Log TSH (z)	Total indirect, levothyroxine users (*n* = 209)	0.151 (0.078 to 0.222)	Sensitivity
Log TSH (z)	Total association, no levothyroxine (*n* = 151)	0.240 (0.142 to 0.348)	Sensitivity
Log TSH (z)	Total indirect, no levothyroxine (*n* = 151)	0.109 (0.051 to 0.177)	Sensitivity
Log TSH (z)	Total association, excluding the highest observed value (*n* = 359)	0.188 (0.103 to 0.268)	Sensitivity
Log TSH (z)	Total indirect, excluding the highest observed value (*n* = 359)	0.138 (0.090 to 0.186)	Sensitivity
Free T4 (z)	Total association	−0.200 (−0.278 to −0.120)	Primary
Free T4 (z)	Total indirect	−0.146 (−0.199 to −0.098)	Primary
Free T4 (z)	Indirect via DERS-16	−0.100 (−0.139 to −0.062)	Primary
Free T4 (z)	Indirect via ERSQ	−0.045 (−0.076 to −0.021)	Primary
Free T4 (z)	Direct association	−0.054 (−0.135 to 0.023)	Primary
Free T4 (z)	Total association, levothyroxine users (*n* = 209)	−0.179 (−0.286 to −0.068)	Sensitivity
Free T4 (z)	Total indirect, levothyroxine users (*n* = 209)	−0.143 (−0.211 to −0.073)	Sensitivity
Free T4 (z)	Total association, no levothyroxine (*n* = 151)	−0.209 (−0.329 to −0.094)	Sensitivity
Free T4 (z)	Total indirect, no levothyroxine (*n* = 151)	−0.140 (−0.219 to −0.067)	Sensitivity
Free T4 (z)	Total association, excluding the highest observed value (*n* = 359)	−0.198 (−0.279 to −0.116)	Sensitivity
Free T4 (z)	Total indirect, excluding the highest observed value (*n* = 359)	−0.143 (−0.192 to −0.096)	Sensitivity
Free T3 (z)	Total association	−0.133 (−0.209 to −0.050)	Sensitivity
Free T3 (z)	Total indirect	−0.086 (−0.130 to −0.044)	Sensitivity

Estimates are standardized association coefficients from parallel two-mediator models adjusted for gestational age, maternal age, and obstetric and socioeconomic covariates (Section 2.4), with bias-corrected 95% confidence intervals from 5000 bootstrap resamples. Confidence intervals are reported for every row, including the stratified and sensitivity rows. The formal exposure-by-levothyroxine interaction is reported in Section 3.5. CI, confidence interval; TSH, thyroid-stimulating hormone; free T4, free thyroxine; anti-TPO, anti-thyroid-peroxidase.

**Table 7:** Index of moderated mediation for gestational week and thyroid autoimmunity, by mediating route.

Moderator	Mediating Route	Index of Moderated Mediation (95% CI)	Raw *p*	Adjusted *p* (BH)
Gestational week (per SD)	DERS-16 difficulties	0.047 (0.020 to 0.083)	<0.001	0.002
Gestational week (per SD)	ERSQ skills	0.017 (−0.004 to 0.045)	0.136	0.182
Gestational week (per SD)	Difference (DERS-16 minus ERSQ)	0.030 (−0.011 to 0.070)	—	—
Anti-TPO ≥ 60 U/mL (binary)	DERS-16 difficulties	−0.014 (−0.044 to 0.013)	0.376	0.376
Anti-TPO ≥ 60 U/mL (binary)	ERSQ skills	−0.027 (−0.053 to −0.008)	0.006	0.013
Anti-TPO ≥ 60 U/mL (binary)	Difference (DERS-16 minus ERSQ)	0.013 (−0.024 to 0.047)	—	—

Indices are from the free T4 exposure models, with bias-corrected bootstrap 95% confidence intervals from 5000 resamples and Benjamini–Hochberg adjusted *p*-values computed across the family of four route-specific indices. Bootstrap *p*-values were obtained as twice the smaller of the proportions of resampled indices at or below and at or above zero. Both moderators are expressed per standard deviation. Difference rows contrast the two route-specific indices for the same moderator and were computed within each bootstrap resample, so the difference intervals account for the covariance between the two indices. Neither difference excluded zero, and neither did the corresponding percentile interval. The observation that one route-specific index excluded zero while the other did not is therefore not evidence that the two routes differ, and all route-specific interpretations are exploratory. For the free T4 exposure, negative indices indicate that the (negative) indirect association became larger in magnitude as the moderator increased. In the bootstrap Johnson–Neyman grid the difficulties-route conditional indirect association remained statistically supported from the earliest sampled week to approximately 30 weeks, with a bootstrap 95% confidence interval for that boundary of about 26 to 38 weeks. Because two-thirds of the anti-thyroid-peroxidase results lay below the lower limit of the measuring interval, where quantitative reporting is not validated, the antibody moderator is defined here as a binary variable at the manufacturer decision threshold; sensitivity of the index to the coding of that variable is reported in Section 3.5 and Table S2, where seven codings are compared. CI, confidence interval; anti-TPO, anti-thyroid-peroxidase.

## Data Availability

The analysis code that produced all reported estimates, including the moderated-mediation engine, the index-difference contrast and the bootstrap Johnson–Neyman procedure, is openly archived in the Zenodo repository at https://doi.org/10.5281/zenodo.21914367 [60] and is also supplied with this submission as a code archive (Supplementary Materials, File S1). The de-identified individual-level dataset is available from the corresponding author on reasonable request, subject to institutional ethics approval and privacy restrictions; because the dataset combines thyroid biochemistry, obstetric data and item-level responses to a depression screen including a self-harm item, its open deposition requires an amendment to the existing ethics approval, which the authors intend to seek.

## Supplementary Materials

The following supporting information can be downloaded at https://www.mdpi.com/article/10.3390/healthcare14172700/s1, Table S1: STROBE checklist for cross-sectional studies mapped to the manuscript; Table S2: Index of moderated mediation under seven codings of the anti-thyroid-peroxidase variable, with stratified re-estimation and the distribution against the assay limits; Table S3: Precision of the design. File S1: Analysis code archive (Python scripts and documentation reproducing all reported estimates).

## References

[1] Bode H., Ivens B., Bschor T., Schwarzer G., Henssler J., Baethge C. (2021). Association of Hypothyroidism and Clinical Depression: A Systematic Review and Meta-Analysis. JAMA Psychiatry.

[2] Alexander E.K., Pearce E.N., Brent G.A., Brown R.S., Chen H., Dosiou C., Grobman W.A., Laurberg P., Lazarus J.H., Mandel S.J. (2017). 2017 Guidelines of the American Thyroid Association for the Diagnosis and Management of Thyroid Disease During Pregnancy and the Postpartum. Thyroid.

[3] Bauer M., Goetz T., Glenn T., Whybrow P.C. (2008). The Thyroid-Brain Interaction in Thyroid Disorders and Mood Disorders. J. Neuroendocrinol..

[4] Korevaar T.I.M., Medici M., Visser T.J., Peeters R.P. (2017). Thyroid Disease in Pregnancy: New Insights in Diagnosis and Clinical Management. Nat. Rev. Endocrinol..

[5] Kuijpens J.L., Vader H.L., Drexhage H.A., Wiersinga W.M., van Son M.J., Pop V.J. (2001). Thyroid Peroxidase Antibodies During Gestation Are a Marker for Subsequent Depression Postpartum. Eur. J. Endocrinol..

[6] Sileo F., Osinga J.A.J., Visser W.E., Jansen T.A., Bramer W.M., Derakhshan A., Citterio V., Tiemeier H., Persani L., Korevaar T.I.M. (2023). Association of Gestational Thyroid Function and Thyroid Peroxidase Antibody Positivity with Postpartum Depression: A Prospective Cohort Study and Systematic Literature Review with Meta-Analysis. Eur. J. Endocrinol..

[7] Bliddal S., Derakhshan A., Xiao Y., Chen L.-M., Männistö T., Ashoor G., Tao F., Brown S.J., Vafeiadi M., Itoh S. (2022). Association of Thyroid Peroxidase Antibodies and Thyroglobulin Antibodies with Thyroid Function in Pregnancy: An Individual Participant Data Meta-Analysis. Thyroid.

[8] Minaldi E., D’Andrea S., Castellini C., Martorella A., Francavilla F., Francavilla S., Barbonetti A. (2020). Thyroid Autoimmunity and Risk of Post-Partum Depression: A Systematic Review and Meta-Analysis of Longitudinal Studies. J. Endocrinol. Investig..

[9] Gross J.J. (1998). The Emerging Field of Emotion Regulation: An Integrative Review. Rev. Gen. Psychol..

[10] Aldao A., Nolen-Hoeksema S., Schweizer S. (2010). Emotion-Regulation Strategies Across Psychopathology: A Meta-Analytic Review. Clin. Psychol. Rev..

[11] Gratz K.L., Roemer L. (2004). Multidimensional Assessment of Emotion Regulation and Dysregulation: Development, Factor Structure, and Initial Validation of the Difficulties in Emotion Regulation Scale. J. Psychopathol. Behav. Assess..

[12] Penner F., Rutherford H.J.V. (2022). Emotion Regulation During Pregnancy: A Call to Action for Increased Research, Screening, and Intervention. Arch. Women’s Ment. Health.

[13] Dikmen-Yildiz P., Ayers S., Phillips L. (2017). Depression, Anxiety, PTSD and Comorbidity in Perinatal Women in Turkey: A Longitudinal Population-Based Study. Midwifery.

[14] Gölbaşı Z., Kelleci M., Kısacık G., Çetin A. (2010). Prevalence and Correlates of Depression in Pregnancy Among Turkish Women. Matern. Child Health J..

[15] Karcaaltincaba D., Ozek M.A., Ocal N., Calis P., Altug Inan M., Bayram M. (2020). Prevalences of Subclinical and Overt Hypothyroidism with Universal Screening in Early Pregnancy. Arch. Gynecol. Obstet..

[16] von Elm E., Altman D.G., Egger M., Pocock S.J., Gøtzsche P.C., Vandenbroucke J.P., STROBE Initiative (2007). The Strengthening the Reporting of Observational Studies in Epidemiology (STROBE) Statement: Guidelines for Reporting Observational Studies. Lancet.

[17] American College of Obstetricians and Gynecologists (2017). Committee Opinion No. 700: Methods for Estimating the Due Date. Obstet. Gynecol..

[18] Korevaar T.I.M., Leung A.M., Alexander E.K., Bliddal S., Boelaert K., Brenta G., Chou R., Dhillon-Smith R., Dosiou C., Eaton J.L. (2026). American Thyroid Association 2026 Guidelines for Thyroid Disease in Preconception, Pregnancy, and Postpartum. Thyroid.

[19] Bjureberg J., Ljótsson B., Tull M.T., Hedman E., Sahlin H., Lundh L.-G., Bjärehed J., DiLillo D., Messman-Moore T., Gumpert C.H. (2016). Development and Validation of a Brief Version of the Difficulties in Emotion Regulation Scale: The DERS-16. J. Psychopathol. Behav. Assess..

[20] Grant M., Salsman N.L., Berking M. (2018). The Assessment of Successful Emotion Regulation Skills Use: Development and Validation of an English Version of the Emotion Regulation Skills Questionnaire. PLoS ONE.

[21] Berking M., Znoj H. (2008). Entwicklung und Validierung eines Fragebogens zur standardisierten Selbsteinschätzung emotionaler Kompetenzen (SEK-27). Z. Psychiatr. Psychol. Psychother..

[22] Cox J.L., Holden J.M., Sagovsky R. (1987). Detection of Postnatal Depression. Development of the 10-Item Edinburgh Postnatal Depression Scale. Br. J. Psychiatry.

[23] Bergink V., Kooistra L., Lambregtse-van den Berg M.P., Wijnen H., Bunevicius R., van Baar A.L., Pop V.J.M. (2011). Validation of the Edinburgh Depression Scale During Pregnancy. J. Psychosom. Res..

[24] Levis B., Negeri Z., Sun Y., Benedetti A., Thombs B.D. (2020). DEPRESsion Screening Data (DEPRESSD) EPDS Group. Accuracy of the Edinburgh Postnatal Depression Scale (EPDS) for Screening to Detect Major Depression Among Pregnant and Postpartum Women: Systematic Review and Meta-Analysis of Individual Participant Data. BMJ.

[25] Huizink A.C., Delforterie M.J., Scheinin N.M., Tolvanen M., Karlsson L., Karlsson H. (2016). Adaption of Pregnancy Anxiety Questionnaire-Revised for All Pregnant Women Regardless of Parity: PRAQ-R2. Arch. Women’s Ment. Health.

[26] Brunton R.J., Dryer R., Saliba A., Kohlhoff J. (2019). The Initial Development of the Pregnancy-Related Anxiety Scale. Women Birth.

[27] Green J.M., Kafetsios K., Statham H.E., Snowdon C.M. (2003). Factor Structure, Validity and Reliability of the Cambridge Worry Scale in a Pregnant Population. J. Health Psychol..

[28] Günay E.Y., Gül A. (2015). Reliability and Validity of the Cambridge Worry Scale in Pregnant Turkish Women. Midwifery.

[29] Yiğit İ., Guzey Yiğit M. (2019). Psychometric Properties of Turkish Version of Difficulties in Emotion Regulation Scale-Brief Form (DERS-16). Curr. Psychol..

[30] Vatan S., Oruçlular Kahya Y. (2018). Turkish Adaptation of Emotion Regulation Skills Questionnaire: The Study of Reliability and Validity. Anatol. J. Psychiatry.

[31] Aydın N., İnandı T., Yiğit A., Şahin Hodoğlugil N.N. (2004). Validation of the Turkish Version of the Edinburgh Postnatal Depression Scale Among Women Within Their First Postpartum Year. Soc. Psychiatry Psychiatr. Epidemiol..

[32] Aksoy Derya Y., Timur Taşhan S., Duman M., Durgun Özan Y. (2018). Turkish Adaptation of the Pregnancy-Related Anxiety Questionnaire-Revised 2: Validity and Reliability Study in Multiparous and Primiparous Pregnancy. Midwifery.

[33] Şolt Kırca A., Kanza Gül D. (2020). Pregnancy-Related Anxiety Scale (PrAS): Validity and Reliability Study of Its Turkish Version. Celal Bayar Univ.-Health Sci. Inst. J..

[34] Gibson J., McKenzie-McHarg K., Shakespeare J., Price J., Gray R. (2009). A Systematic Review of Studies Validating the Edinburgh Postnatal Depression Scale in Antepartum and Postpartum Women. Acta Psychiatr. Scand..

[35] MacKinnon D.P., Lockwood C.M., Williams J. (2004). Confidence Limits for the Indirect Effect: Distribution of the Product and Resampling Methods. Multivar. Behav. Res..

[36] Benjamini Y., Hochberg Y. (1995). Controlling the False Discovery Rate: A Practical and Powerful Approach to Multiple Testing. J. R. Stat. Soc. Ser. B Stat. Methodol..

[37] VanderWeele T.J., Ding P. (2017). Sensitivity Analysis in Observational Research: Introducing the E-Value. Ann. Intern. Med..

[38] Preacher K.J., Rucker D.D., Hayes A.F. (2007). Addressing Moderated Mediation Hypotheses: Theory, Methods, and Prescriptions. Multivar. Behav. Res..

[39] Hayes A.F. (2015). An Index and Test of Linear Moderated Mediation. Multivar. Behav. Res..

[40] Preacher K.J., Curran P.J., Bauer D.J. (2006). Computational Tools for Probing Interactions in Multiple Linear Regression, Multilevel Modeling, and Latent Curve Analysis. J. Educ. Behav. Stat..

[41] Hoenig J.M., Heisey D.M. (2001). The Abuse of Power: The Pervasive Fallacy of Power Calculations for Data Analysis. Am. Stat..

[42] Levine M., Ensom M.H.H. (2001). Post Hoc Power Analysis: An Idea Whose Time Has Passed?. Pharmacotherapy.

[43] Helsel D.R. (2006). Fabricating Data: How Substituting Values for Nondetects Can Ruin Results, and What Can Be Done about It. Chemosphere.

[44] Liu Y., Osinga J.A.J., Feldt-Rasmussen U., Vrijkotte T.G.M., Taylor P.N., Aminorroaya A., Ashoor G., Bliddal S., Chen L.-M., Vaidya B. (2025). Interpretation of the Association Between Thyroid Peroxidase Antibodies and Thyroid Function During Pregnancy: An Individual Participant Data Meta-Analysis. J. Autoimmun..

[45] Verhelst P., Sels L., Lemmens G., Verhofstadt L. (2024). The Role of Emotion Regulation in Perinatal Depression and Anxiety: A Systematic Review. BMC Psychol..

[46] Weinmar F., Fransson E., Derntl B., Skalkidou A. (2025). Emotion Regulation Is Robustly Associated with Perinatal Depressive Symptoms in a Swedish National Cohort. Nat. Ment. Health.

[47] Gross J.J. (2015). Emotion Regulation: Current Status and Future Prospects. Psychol. Inq..

[48] Okosieme O.E., Agrawal M., Usman D., Evans C. (2021). Method-Dependent Variation in TSH and FT4 Reference Intervals in Pregnancy: A Systematic Review. Ann. Clin. Biochem..

[49] Osinga J.A.J., Derakhshan A., Palomaki G.E., Ashoor G., Männistö T., Maraka S., Chen L., Bliddal S., Lu X., Taylor P.N. (2022). TSH and FT4 Reference Intervals in Pregnancy: A Systematic Review and Individual Participant Data Meta-Analysis. J. Clin. Endocrinol. Metab..

[50] Schmidt M., Voell M., Rahlff I., Dietlein M., Kobe C., Faust M., Schicha H. (2008). Long-Term Follow-Up of Antithyroid Peroxidase Antibodies in Patients with Chronic Autoimmune Thyroiditis (Hashimoto’s Thyroiditis) Treated with Levothyroxine. Thyroid.

[51] Levie D., Korevaar T.I.M., Bath S.C., Dalmau-Bueno A., Murcia M., Espada M., Dineva M., Ibarluzea J.M., Sunyer J., Tiemeier H. (2018). Thyroid Function in Early Pregnancy, Child IQ, and Autistic Traits: A Meta-Analysis of Individual Participant Data. J. Clin. Endocrinol. Metab..

[52] Holbanel L.-M., Dragota R.S., Popescu M., Glavan D.G., Pirlog M.C., Turcu-Stiolica A. (2026). No Concurrent Association Found Between Maternal Thyroid Hormone Concentrations (TSH, FT4, FT3) and Antepartum Depression in Late Pregnancy: A Meta-Analysis Highlighting the Need for Categorical Risk Assessment. Psychiatry Int..

[53] Casey B.M., Thom E.A., Peaceman A.M., Varner M.W., Sorokin Y., Hirtz D.G., Reddy U.M., Wapner R.J., Thorp J.M., Saade G. (2017). Treatment of Subclinical Hypothyroidism or Hypothyroxinemia in Pregnancy. N. Engl. J. Med..

[54] Lazarus J.H., Bestwick J.P., Channon S., Paradice R., Maina A., Rees R., Chiusano E., John R., Guaraldo V., George L.M. (2012). Antenatal Thyroid Screening and Childhood Cognitive Function. N. Engl. J. Med..

[55] Maxwell S.E., Cole D.A. (2007). Bias in Cross-Sectional Analyses of Longitudinal Mediation. Psychol. Methods.

[56] Tan H.-L., Ge H., Qin Z.-E., Jiang Y.-L., Chang S., Tang N. (2025). Evaluating the Diagnostic Efficiency of Ultrasound and Serum Autoantibodies in Hashimoto’s Thyroiditis: A Cross-Sectional Study. Sci. Rep..

[57] Podsakoff P.M., MacKenzie S.B., Lee J.-Y., Podsakoff N.P. (2003). Common Method Biases in Behavioral Research: A Critical Review of the Literature and Recommended Remedies. J. Appl. Psychol..

[58] Siemsen E., Roth A., Oliveira P. (2010). Common Method Bias in Regression Models with Linear, Quadratic, and Interaction Effects. Organ. Res. Methods.

[59] Vural M., Koc E., Evliyaoglu O., Acar H.C., Aydin A.F., Kucukgergin C., Apaydin G., Erginoz E., Babazade X., Sharifova S. (2021). Iodine Status of Turkish Pregnant Women and Their Offspring: A National Cross-Sectional Survey. J. Trace Elem. Med. Biol..

[60] Misirlioglu R., Yarsilikal Guleroglu F., Cetin A. (2026). Analysis Code for: Emotion-Regulation Difficulties and Skills in the Cross-Sectional Association Between Thyroid Function and Antenatal Distress in Hypothyroid Pregnancy [Computer Software]. Zenodo.

