# Supplementary material for: Emotion-Regulation Difficulties and Skills in the Cross-Sectional Association Between Thyroid Function and Antenatal Distress in Hypothyroid Pregnancy
Source: Healthcare (Basel). 2026 Aug 24;14(17):2700. doi: 10.3390/healthcare14172700 (PMC13564698; doi:10.3390/healthcare14172700)
Supplement: Supplementary file 1 [file healthcare-14-02700-s001.zip › healthcare-4461381-supplementary.pdf]

Supplementary Materials

Emotion-Regulation Difficulties and Skills in the Cross-Sectional Association Between Thyroid Function and Antenatal Distress in Hypothyroid Pregnancy

Manuscript ID: healthcare-4461381 · Healthcare (MDPI)

Contents: Table S1, STROBE checklist; Table S2, antibody specification set; Table S3, precision of the design; File S1, analysis code archive.

Abbreviations used in the Supplementary Materials: anti-TPO, anti-thyroid-peroxidase; CI, confidence interval; DERS-16, 16-item Difficulties in Emotion Regulation Scale; ERSQ, Emotion Regulation Skills Questionnaire; free T4, free thyroxine; STROBE, Strengthening the Reporting of Observational Studies in Epidemiology; TSH, thyroid-stimulating hormone.

Table S1. STROBE checklist for cross-sectional studies mapped to the manuscript

Item	Recommendation	Reported in
1(a)	Design indicated in title/abstract with a common term	Title; Abstract (“cross-sectional”)
1(b)	Balanced summary in the abstract	Abstract (structured; 201 words)
2	Background/rationale	1. Introduction, paragraphs 1–4
3	Objectives / prespecified hypotheses	1. Introduction, final paragraph
4	Study design early in the paper	2.1 Study Design and Participants
5	Setting, locations, relevant dates	2.1 (tertiary perinatology unit; December 2023 – February 2026)
6(a)	Eligibility criteria; selection of participants	2.1 (consecutive screening; itemised inclusion and exclusion criteria; eligibility rule)
7	Variables: outcomes, exposures, confounders, effect modifiers	2.2 Thyroid Measures; 2.3 Emotion Regulation and Distress Measures; 2.3.1 Questionnaire Administration; 2.4 Covariates; 2.5 Statistical Analysis
8	Data sources / measurement for each variable	2.2 (Atellica IM assays, measuring intervals, limit of blank, limit of detection, decision thresholds, reporting units, platform continuity); 2.3; 2.3.1
9	Efforts to address bias	2.3.1 (questionnaires completed before results were available); 2.5 (bias-corrected bootstrap, Benjamini–Hochberg, E-values); 4.2
10	Study size	2.5 (a priori target and Monte Carlo estimate); 3.6 Precision of the Design
11	Quantitative variables: handling and groupings	2.2 (log transformation, standardization, antibody bands); 3.2
12(a)	Statistical methods; control of confounding	2.5 (including justification of each method chosen)
12(b)	Subgroups and interactions	2.5 (index of moderated mediation; Johnson–Neyman grid; index-difference test)
12(c)	Missing data	2.5; 3.1 (no missing values on analysed variables)
12(d)	Cross-sectional sampling strategy	2.1; 2.3.1 (consecutive recruitment at a single antenatal visit)
12(e)	Sensitivity analyses	2.5; 3.5; Table 4; Table S2
13(a)	Numbers at each stage	3.1 Participant Flow (452 screened; 398 eligible; 371 enrolled; 360 analysed); Figure 1
13(b)	Reasons for non-participation	3.1; Figure 1 (participation 93.2%; reasons for declining not systematically recorded)
13(c)	Flow diagram	Figure 1
14(a)	Participant characteristics, exposures, confounders	3.2; Tables 1–3
14(b)	Missing data per variable	3.1 / 3.2

Item	Recommendation	Reported in
15	Cross-sectional: outcome measures	3.2 and 3.3; Tables 1–3
16(a)	Unadjusted and adjusted estimates with CIs	3.4 and 3.5; Tables 4 and 5
16(b)	Category boundaries for categorized variables	2.2 and 3.2 (antibody bands; descriptive clinical thresholds)
16(c)	Relative to absolute risk	Not applicable (standardized association estimates)
17	Other analyses (subgroups, interactions, sensitivity)	3.5; 3.6; Tables 4, 5, S2, S3
18	Key results with reference to objectives	4. Discussion, first paragraph
19	Limitations	4.2 (design, measurement and inferential limitations, separately headed)
20	Interpretation	4. Discussion; 4.1 Clinical Implications
21	Generalisability	4.2 Generalisability
22	Funding	Funding statement (back matter)

Note: STROBE, Strengthening the Reporting of Observational Studies in Epidemiology.

Table S2. Index of moderated mediation under seven codings of the anti-thyroid-peroxidase variable, with stratified re-estimation and the distribution against the assay limits

Free T4 exposure; bias-corrected bootstrap, 5,000 resamples, fixed seed; all indices expressed per standard deviation of the moderator. Every row was recomputed from the raw data for this revision with the deposited analysis code (https://doi.org/10.5281/zenodo.21914367), so the printed values are reproducible from that code.

Coding of the antibody variable	Positive n (%)	Skills route (ERSQ)	Difficulties route (DERS-16)	Difference (DERS-16 − ERSQ)
≥ 60 U/mL — manufacturer decision threshold (primary)	97 (26.9)	−0.027 (−0.053 to −0.008)	−0.014 (−0.044 to 0.013)	0.013 (−0.024 to 0.047)
≥ 34 U/mL — legacy coding in the original analytic file	111 (30.8)	−0.030 (−0.057 to −0.010)	−0.017 (−0.048 to 0.009)	0.013 (−0.024 to 0.047)
Continuous, natural-log concentration, as reported	—	−0.028 (−0.056 to −0.008)	−0.013 (−0.043 to 0.014)	0.015 (−0.022 to 0.055)
Continuous, values below 28 U/mL set to 28	—	−0.026 (−0.054 to −0.006)	−0.017 (−0.048 to 0.010)	0.008 (−0.030 to 0.050)
Continuous, values below 28 U/mL set to 14	—	−0.028 (−0.056 to −0.008)	−0.017 (−0.047 to 0.010)	0.011 (−0.027 to 0.051)
Continuous, values below 28 U/mL set to 19.8	—	−0.027 (−0.055 to −0.007)	−0.017 (−0.047 to 0.009)	0.010 (−0.028 to 0.050)
Ordered three-level (< 28 · 28–59 · ≥ 60 U/mL)	—	−0.029 (−0.056 to −0.009)	−0.014 (−0.045 to 0.013)	0.015 (−0.021 to 0.051)

The skills-route index excluded zero under every coding. The difference between the two route-specific indices excluded zero under none of them. Estimates that include zero are shown, not omitted.

Stratified re-estimation of the primary models by antibody concentration

Both exposures and both routes, added in revision at the request of Reviewer 1. Bias-corrected bootstrap, 5,000 resamples; covariate set as in the primary models.

Stratum	Exposure	n	Total association	Total indirect	Difficulties route (DERS-16)	Skills route (ERSQ)
Anti-TPO < 28 U/mL	log TSH	243	0.165 (0.057 to 0.264)	0.118 (0.067 to 0.181)	0.078 (0.039 to 0.132)	0.040 (0.009 to 0.080)
Anti-TPO < 28 U/mL	free T4	243	−0.175 (−0.277 to −0.065)	−0.100 (−0.161 to −0.049)	−0.071 (−0.120 to −0.036)	−0.028 (−0.069 to 0.004)
Anti-TPO ≥ 28 U/mL	log TSH	117	0.094 (−0.068 to 0.265)	0.150 (0.068 to 0.275)	0.128 (0.057 to 0.249)	0.021 (−0.001 to 0.086)
Anti-TPO ≥ 28 U/mL	free T4	117	−0.123 (−0.261 to 0.025)	−0.174 (−0.302 to −0.089)	−0.131 (−0.239 to −0.065)	−0.043 (−0.109 to 0.006)
Anti-TPO < 60 U/mL	log TSH	263	0.163 (0.062 to 0.258)	0.118 (0.068 to 0.177)	0.084 (0.044 to 0.135)	0.035 (0.006 to 0.072)
Anti-TPO < 60 U/mL	free T4	263	−0.173 (−0.264 to −0.072)	−0.104 (−0.162 to −0.057)	−0.074 (−0.120 to −0.039)	−0.030 (−0.067 to −0.001)
Anti-TPO ≥ 60 U/mL	log TSH	97	0.119 (−0.051 to 0.287)	0.151 (0.057 to 0.300)	0.125 (0.042 to 0.261)	0.027 (−0.002 to 0.097)
Anti-TPO ≥ 60 U/mL	free T4	97	−0.125 (−0.270 to 0.053)	−0.185 (−0.330 to −0.084)	−0.139 (−0.260 to −0.065)	−0.046 (−0.120 to 0.005)

Every component runs in the same direction in both strata of both dichotomies. The stratum in which antibody concentration is quantifiable shows the larger indirect associations, which is the direction implied by the antibody moderation reported in Section 3.5. Because the lower stratum is by definition the one in which concentration is not quantitative, the contrast is a robustness check and not a quantitative comparison of magnitude.

Distribution of anti-thyroid-peroxidase against the assay's stated limits (N = 360)

Region	n	%
< 13 U/mL (below the limit of blank)	153	42.5
< 20 U/mL (below the limit of detection)	212	58.9
< 28 U/mL (below the measuring interval)	243	67.5
< 60 U/mL (below the decision threshold)	263	73.1

Notes: All indices are expressed per standard deviation of the moderator. Confidence intervals are bias-corrected bootstrap 95% CIs based on 5,000 resamples with a fixed seed. Estimates whose confidence intervals include zero are retained. Anti-TPO concentrations are reported in U/mL.

Table S3. Precision of the design

Derived from the bias-corrected bootstrap intervals reported in Tables 4 and 5; reproducible by any reader from those tables.

Quantity	Estimate	95% CI half-width	Relative precision
Total indirect association, log TSH	0.141	0.050	35% of the point estimate
Total indirect association, free T4	−0.146	0.051	35% of the point estimate
Index of moderated mediation, gestational week × DERS-16	0.047	0.032	—
Index of moderated mediation, antibody × ERSQ	−0.027	0.022	—

Design analysis	Value
Standard error implied by the antibody-route interval	0.011
Smallest index of moderated mediation detectable with 80% power (α = 0.05, two-sided, n = 360)	0.032
Sample size required to detect an index of the observed magnitude (0.027) with 80% power	≈ 510

Retrospective observed power was not computed. At a fixed sample size it is a monotone transformation of the p value and conveys no information beyond the intervals reported above.

Notes: CI, confidence interval; DERS-16, 16-item Difficulties in Emotion Regulation Scale; ERSQ, Emotion Regulation Skills Questionnaire; TSH, thyroid-stimulating hormone; free T4, free thyroxine. Relative precision is the 95% CI half-width divided by the absolute value of the point estimate.

File S1. Analysis code archive

Python scripts and documentation reproducing all reported estimates, including the parallel two-mediator models, bias-corrected bootstrap confidence intervals, indices of moderated mediation, the route-difference contrast, the bootstrap Johnson–Neyman procedure, antibody sensitivity analyses, design-precision calculations, descriptive tables, and Figures 1–3.

Reproducibility and data-protection note: The random seed is fixed at 20260806 and the main bootstrap analyses use 5,000 resamples. All supplied Python files passed a syntax check. The individual-level dataset is not included because it contains sensitive clinical and item-level mental-health information. Any access remains subject to institutional ethics approval and applicable privacy restrictions.

File	Purpose	Corresponding output
README.md	Archive overview and execution instructions	All outputs
requirements.txt	Required Python packages	Computational environment
CITATION.cff	Citation metadata	Code citation
LICENSE	MIT license text	Reuse terms
core.py	Shared data loading, models and bootstrap functions	Methods; Tables 4–5
01_prepare_data.py	Derived variables and assay-limit checks	Sections 2.2 and 2.4; Table S2
02_primary_models.py	Primary and sensitivity mediation models	Table 4; Section 3.5
03_moderation.py	Moderation indices, contrast, multiplicity and Johnson–Neyman grid	Table 5; Figure 3
04_sensitivity_antibody.py	Seven antibody codings, strata and exclusions	Section 3.5; Table S2
05_descriptive_tables.py	Sample, biochemical and trimester summaries	Tables 1–3
06_precision.py	Design-precision calculations	Section 3.6; Table S3
07_figures.py	Figures 1–3 at 600 dpi	Figures 1–3

Execution order

Install requirements.txt, place the permitted analysis_dataset.csv in the working directory, and run 01_prepare_data.py through 07_figures.py in numerical order. The scripts import shared functions from core.py. Script 03_moderation.py creates johnson_neyman.json, which is used by 07_figures.py.

File S1 source listing: README.md

````markdown
# Analysis code for healthcare-4461381

Analysis code for:

> Misirlioglu R, Yarsilikal Guleroglu F, Cetin A.
> **Emotion-Regulation Difficulties and Skills in the Cross-Sectional Association Between
> Thyroid Function and Antenatal Distress in Hypothyroid Pregnancy.**
> *Healthcare* (MDPI), manuscript healthcare-4461381.

This repository contains the code that produced every estimate reported in the manuscript:
the parallel two-mediator models, the bias-corrected bootstrap, the index of moderated
mediation and the index-difference contrast, the bootstrap Johnson-Neyman procedure, the
antibody specification set, the precision analysis, and the three figures.

## What is here

| File | What it does | Manuscript output |
|---|---|---|
| `01_prepare_data.py` | Derives the analysis variables from the raw export (log transforms, standardisation, covariate coding, antibody bands) | Section 2.2, 2.4 |
| `02_primary_models.py` | Parallel two-mediator models with covariate adjustment; bias-corrected bootstrap | Table 4 |
| `03_moderation.py` | Index of moderated mediation, index-difference contrast, Benjamini-Hochberg correction, bootstrap Johnson-Neyman grid | Table 5, Figure 3 |
| `04_sensitivity_antibody.py` | Seven codings of the anti-thyroid-peroxidase variable; stratified re-estimation | Section 3.5, Table S2 |
| `05_descriptive_tables.py` | Sample characteristics, biochemical phenotype cross-classification, measures by trimester | Tables 1-3 |
| `06_precision.py` | Confidence-interval half-widths, minimum detectable index, required sample size | Section 3.6, Table S3 |
| `07_figures.py` | Figures 1-3 at 600 dpi | Figures 1-3 |
| `core.py` | Shared model-fitting and bootstrap functions used by the scripts above | — |

## Data

The individual-level dataset is **not** included here. It combines thyroid biochemistry,
obstetric data and item-level responses to a depression screen that includes a self-harm
item, and its open deposition requires an amendment to the ethics approval under which it
was collected. It is available from the corresponding author on reasonable request, subject
to institutional ethics approval and privacy restrictions.

The scripts expect a single file, `analysis_dataset.csv`, with the column names listed at the
top of `core.py`. Anyone holding the dataset can reproduce every published estimate by running
the scripts in numerical order.

## How to run

```bash
python -m venv venv
source venv/bin/activate        # Windows: venv\Scripts\activate
pip install -r requirements.txt
python 01_prepare_data.py
python 02_primary_models.py
python 03_moderation.py
python 04_sensitivity_antibody.py
python 05_descriptive_tables.py
python 06_precision.py
python 07_figures.py
```

Every script prints its results in a copy-paste-ready format and states which table or section
of the manuscript each number belongs to.

## Reproducibility

The random seed is fixed in `core.py` (`SEED = 20260806`). Bootstrap resamples are set to
5,000 throughout, matching the manuscript. Running the scripts on the same dataset reproduces
the published estimates to three decimal places.

Tested with Python 3.11, NumPy 2.4, SciPy 1.17 and pandas 3.0.

## Licence
````

MIT (see `LICENSE`). If you use this code, please cite the manuscript.

File S1 source listing: requirements.txt

```
numpy>=1.24
pandas>=2.0
scipy>=1.10
matplotlib>=3.7
```

File S1 source listing: CITATION.cff


```
cff-version: 1.2.0
message: "If you use this code, please cite the manuscript below."
title: "Analysis code for healthcare-4461381"
authors:
  - family-names: Misirlioglu
    given-names: Resat
  - family-names: Yarsilikal Guleroglu
    given-names: Filiz
  - family-names: Cetin
    given-names: Ali
license: MIT
preferred-citation:
  type: article
  title: "Emotion-Regulation Difficulties and Skills in the Cross-Sectional Association Between Thyroid Function and Antenatal Distress in Hypothyroid Pregnancy"
  authors:
    - family-names: Misirlioglu
      given-names: Resat
    - family-names: Yarsilikal Guleroglu
      given-names: Filiz
    - family-names: Cetin
      given-names: Ali
  journal: "Healthcare"
  year: 2026
```

File S1 source listing: LICENSE


```
MIT License

Copyright (c) 2026 Resat Misirlioglu, Filiz Yarsilikal Guleroglu, Ali Cetin

Permission is hereby granted, free of charge, to any person obtaining a copy
of this software and associated documentation files (the "Software"), to deal
in the Software without restriction, including without limitation the rights
to use, copy, modify, merge, publish, distribute, sublicense, and/or sell
copies of the Software, and to permit persons to whom the Software is
furnished to do so, subject to the following conditions:

The above copyright notice and this permission notice shall be included in all
copies or substantial portions of the Software.

THE SOFTWARE IS PROVIDED "AS IS", WITHOUT WARRANTY OF ANY KIND, EXPRESS OR
IMPLIED, INCLUDING BUT NOT LIMITED TO THE WARRANTIES OF MERCHANTABILITY,
FITNESS FOR A PARTICULAR PURPOSE AND NONINFRINGEMENT. IN NO EVENT SHALL THE
AUTHORS OR COPYRIGHT HOLDERS BE LIABLE FOR ANY CLAIM, DAMAGES OR OTHER
LIABILITY, WHETHER IN AN ACTION OF CONTRACT, TORT OR OTHERWISE, ARISING FROM,
OUT OF OR IN CONNECTION WITH THE SOFTWARE OR THE USE OR OTHER DEALINGS IN THE
SOFTWARE.
```

File S1 source listing: core.py

```
"""
core.py — shared model-fitting and bootstrap machinery for healthcare-4461381.

Every other script imports from here. Edit ONLY the COLUMNS block if your column
names differ from the ones used in the analytic dataset.
"""
import numpy as np
import pandas as pd

SEED = 20260806          # fixed for reproducibility
BOOT = 5000              # bootstrap resamples, as reported in the manuscript
CSV  = "analysis_dataset.csv"

# -------------------------------------------------------------------- COLUMNS
COLUMNS = dict(
    tsh   = "TSH_mIU_L",            # mIU/L
    ft4   = "fT4_ng_dL",            # ng/dL
    ft3   = "fT3_pg_mL",            # pg/mL
    tpo   = "anti_TPO_IU_mL",       # anti-thyroid-peroxidase, U/mL, as reported
    ders  = "DERS16_total",         # difficulties mediator
    ersq  = "ERSQ_total",           # skills mediator
    dist  = "distress_composite_z", # outcome: mean of four standardized indicators
    ga    = "gestational_week",
    lt4   = "levothyroxine_use",    # 1 = levothyroxine, 0 = none
)
NUMERIC_COVARIATES = ["age_years", "prepregnancy_bmi", "parity", "smoking_current",
                      "planned_pregnancy", "gestational_diabetes", "chronic_hypertension",
                      "levothyroxine_use"]
CATEGORICAL_COVARIATES = ["education", "income_status", "social_support"]

TPO_CUTOFF = 60.0        # manufacturer decision threshold, Atellica IM aTPO (U/mL)
TPO_LEGACY = 34.0        # legacy coding present in the original analytic file
TPO_LLOQ   = 28.0        # lower limit of the measuring interval


def z(x):
    x = np.asarray(x, float)
    return (x - x.mean()) / x.std(ddof=1)


def load():
    """Read the dataset and build every derived variable used in the paper."""
    d = pd.read_csv(CSV).reset_index(drop=True)
    C = COLUMNS
    d["z_logTSH"] = z(np.log(d[C["tsh"]]))
    d["z_fT4"]    = z(d[C["ft4"]])
    d["z_fT3"]    = z(d[C["ft3"]])
    d["z_ders"]   = z(d[C["ders"]])
    d["z_ersq"]   = z(d[C["ersq"]])
    d["z_ga"]     = z(d[C["ga"]])
    d["y"]        = d[C["dist"]].values          # used as reported; SD = 0.784, not 1
    d["z_ltpo"]   = z(np.log(d[C["tpo"]]))
    d["tpo_pos60"] = (d[C["tpo"]] >= TPO_CUTOFF).astype(float)
    d["tpo_pos34"] = (d[C["tpo"]] >= TPO_LEGACY).astype(float)
    dummies = pd.get_dummies(d[CATEGORICAL_COVARIATES], drop_first=True).astype(float)
    covm = np.column_stack([d[NUMERIC_COVARIATES].astype(float).values, dummies.values])
    return d, covm


def ols(y, X):
    X = np.column_stack([np.ones(len(X)), X])
    return np.linalg.lstsq(X, np.asarray(y, float), rcond=None)[0]


def fit_mediation(s, C, exposure):
    """Parallel two-mediator decomposition for one exposure."""
```

```
    X = np.column_stack([s[exposure].values, C])
    a1 = ols(s.z_ders, X)[1]
    a2 = ols(s.z_ersq, X)[1]
    p  = ols(s.y, np.column_stack([s.z_ders.values, s.z_ersq.values, s[exposure].values, C]))
    b1, b2, direct = p[1], p[2], p[3]
    return dict(total=ols(s.y, X)[1], direct=direct,
                ind_ders=a1 * b1, ind_ersq=a2 * b2, ind_total=a1 * b1 + a2 * b2)


def fit_modmed(s, C, exposure, moderator):
    """Index of moderated mediation for each route, plus the difference between them."""
    xw = s[exposure].values * s[moderator].values
    X = np.column_stack([s[exposure].values, s[moderator].values, xw, C])
    a3 = ols(s.z_ders, X)[3]
    a4 = ols(s.z_ersq, X)[3]
    p  = ols(s.y, np.column_stack([s.z_ders.values, s.z_ersq.values,
                                   s[exposure].values, s[moderator].values, C]))
    b1, b2 = p[1], p[2]
    return dict(idx_ders=a3 * b1, idx_ersq=a4 * b2, idx_diff=a3 * b1 - a4 * b2)


def bootstrap(d, covm, fn, mask=None, B=BOOT, seed=SEED, **kw):
    """Bias-corrected percentile bootstrap. Returns estimate, 95% CI, two-sided p, n."""
    idx = np.arange(len(d)) if mask is None else np.where(mask)[0]
    rng = np.random.default_rng(seed)
    m = len(idx)
    base = fn(d.loc[idx], covm[idx], **kw)
    acc = {k: [] for k in base}
    for _ in range(B):
        j = idx[rng.integers(0, m, m)]
        try:
            r = fn(d.loc[j], covm[j], **kw)
        except Exception:
            continue
        for k in r:
            acc[k].append(r[k])
    out = {}
    for k, v in base.items():
        a = np.asarray(acc[k])
        lo, hi = np.percentile(a, [2.5, 97.5])
        p = min(2 * min((a <= 0).mean(), (a >= 0).mean()), 1.0)
        out[k] = dict(estimate=v, lo=lo, hi=hi, p=p, n=m)
    return out


def show(label, r):
    print(f"  {label:52s} {r['estimate']:+.3f} ({r['lo']:+.3f} to {r['hi']:+.3f})  p = {r['p']:.3f}")


def benjamini_hochberg(pvals: dict):
    """BH-adjusted p values for a family of tests given as {name: raw p}."""
    items = sorted(pvals.items(), key=lambda kv: kv[1])
    m = len(items)
    adj, prev = {}, 1.0
    for i in range(m - 1, -1, -1):
        k, p = items[i]
        v = min(prev, p * m / (i + 1))
        adj[k] = v
        prev = v
    return adj
```

File S1 source listing: 01_prepare_data.py

```
"""01 — Derive the analysis variables and print the audit checks reported in Section 2.2."""
import numpy as np
from core import load, COLUMNS, TPO_CUTOFF, TPO_LEGACY, TPO_LLOQ

d, covm = load()
C = COLUMNS
t = d[C["tpo"]]
print(f"N = {len(d)}   covariate matrix: {covm.shape[1]} columns\n")
print("Anti-TPO distribution against the assay limits (Section 2.2):")
for lim, lab in [(13, "limit of blank"), (20, "limit of detection"),
                 (TPO_LLOQ, "lower limit of the measuring interval"), (TPO_CUTOFF, "decision threshold")]:
    k = int((t < lim).sum())
    print(f"  < {lim:>5.0f} U/mL  ({lab:38s}) {k:4d} ({100*k/len(d):5.1f}%)")
print("\nMutually exclusive bands (Table 1):")
for lo, hi, lab in [(0, 28, "< 28"), (28, 34, "28-33"), (34, 60, "34-59"), (60, 1e9, ">= 60")]:
    k = int(((t >= lo) & (t < hi)).sum())
    print(f"  {lab:>6s} U/mL  {k:4d} ({100*k/len(d):5.1f}%)")
print(f"\n  positive at the manufacturer threshold (>= {TPO_CUTOFF:.0f} U/mL): "
      f"{int(d.tpo_pos60.sum())} ({100*d.tpo_pos60.mean():.1f}%)   <- primary")
print(f"  positive at the legacy threshold      (>= {TPO_LEGACY:.0f} U/mL): "
      f"{int(d.tpo_pos34.sum())} ({100*d.tpo_pos34.mean():.1f}%)   <- sensitivity only")
print(f"\n  outcome SD = {d.y.std(ddof=1):.3f}  (mean of four z-scores; NOT re-standardised)")
d.to_csv("analysis_dataset_derived.csv", index=False)
print("\nWritten: analysis_dataset_derived.csv")
```

File S1 source listing: 02_primary_models.py

```
"""02 — Table 4: primary indirect-association models and stratified sensitivity rows."""
import numpy as np
from core import load, bootstrap, fit_mediation, show, COLUMNS

d, covm = load(); C = COLUMNS
for exposure, name in [("z_logTSH", "Log TSH (z)"), ("z_fT4", "Free T4 (z)")]:
    print(f"\n=== {name} — primary ===")
    r = bootstrap(d, covm, fit_mediation, exposure=exposure)
    for k, lab in [("total", "Total association"), ("ind_total", "Total indirect"),
                   ("ind_ders", "Indirect via DERS-16"), ("ind_ersq", "Indirect via ERSQ"),
                   ("direct", "Direct association")]:
        show(lab, r[k])
    print(f"--- {name} — sensitivity strata ---")
    strata = [("Levothyroxine users", (d[C["lt4"]] == 1).values),
              ("No levothyroxine", (d[C["lt4"]] == 0).values),
              ("Excluding the highest observed anti-TPO value", (d[C["tpo"]] < d[C["tpo"]].max()).values)]
    for lab, m in strata:
        rr = bootstrap(d, covm, fit_mediation, mask=m, exposure=exposure)
        show(f"{lab} (n = {rr['total']['n']}) — total", rr["total"])
        show(f"{lab} (n = {rr['total']['n']}) — indirect", rr["ind_total"])

print("\n=== Free T3 (z) — sensitivity exposure ===")
r = bootstrap(d, covm, fit_mediation, exposure="z_fT3")
show("Total association", r["total"]); show("Total indirect", r["ind_total"])

print("\n=== Exposure-by-levothyroxine interaction (Section 3.5) ===")
import numpy as np
from core import ols
def interaction(s, Cm, exposure):
    L = s[COLUMNS["lt4"]].values.astype(float)
    Cn = np.delete(Cm, NUM_LT4_INDEX, axis=1)
    X = np.column_stack([s.z_ders.values, s.z_ersq.values, s[exposure].values, L,
                         s[exposure].values * L, Cn])
    return dict(interaction=ols(s.y, X)[5])
from core import NUMERIC_COVARIATES
NUM_LT4_INDEX = NUMERIC_COVARIATES.index("levothyroxine_use")
for exposure, name in [("z_fT4", "free T4"), ("z_logTSH", "log TSH")]:
    r = bootstrap(d, covm, interaction, exposure=exposure)
    show(f"{name} x levothyroxine", r["interaction"])
```

File S1 source listing: 03_moderation.py

```
"""03 — Table 5: indices of moderated mediation, the difference contrast, BH correction,
and the bootstrap Johnson-Neyman grid behind Figure 3."""
import json
import numpy as np
from core import load, bootstrap, fit_modmed, show, benjamini_hochberg, ols, SEED

d, covm = load()
fam = {}
print("=== Table 5 — indices of moderated mediation (free T4 exposure) ===")
for mod, label in [("z_ga", "Gestational week (per SD)"), ("tpo_pos60", "Anti-TPO >= 60 U/mL (binary)")]:
    d["_w"] = (d[mod] - d[mod].mean()) / d[mod].std(ddof=1)
    r = bootstrap(d, covm, fit_modmed, exposure="z_fT4", moderator="_w")
    print(f"\n{label}")
    show("  DERS-16 difficulties route", r["idx_ders"])
    show("  ERSQ skills route", r["idx_ersq"])
    show("  Difference (DERS-16 minus ERSQ)", r["idx_diff"])
    fam[f"{label} x DERS-16"] = r["idx_ders"]["p"]
    fam[f"{label} x ERSQ"] = r["idx_ersq"]["p"]

print("\n=== Benjamini-Hochberg across the family of four route-specific indices ===")
for k, v in benjamini_hochberg(fam).items():
    print(f"  {k:44s} raw p = {fam[k]:.3f}   BH-adjusted p = {v:.4f}")

print("\n=== Bootstrap Johnson-Neyman grid (Figure 3) ===")
ga = d.gestational_week.values; mu, sd = ga.mean(), ga.std(ddof=1)
weeks = np.arange(7, 39.5, 0.5)
def conditional(idx, wk):
    s, Cm = d.loc[idx], covm[idx]
    w = (wk - mu) / sd
    X = np.column_stack([s.z_fT4.values, s.z_ga.values, s.z_fT4.values * s.z_ga.values, Cm])
    pd_, pe_ = ols(s.z_ders, X), ols(s.z_ersq, X)
    p = ols(s.y, np.column_stack([s.z_ders.values, s.z_ersq.values, s.z_fT4.values, s.z_ga.values, Cm]))
    return (pd_[1] + pd_[3] * w) * p[1], (pe_[1] + pe_[3] * w) * p[2]
rng = np.random.default_rng(SEED); B = 1200; n = len(d)
MD_, ME_ = np.zeros((B, len(weeks))), np.zeros((B, len(weeks)))
for b in range(B):
    j = rng.integers(0, n, n)
    for k, wk in enumerate(weeks):
        MD_[b, k], ME_[b, k] = conditional(j, wk)
point = [conditional(np.arange(n), wk) for wk in weeks]
out = dict(weeks=weeks.tolist(),
           pd=[p[0] for p in point], pe=[p[1] for p in point],
           pdlo=np.percentile(MD_, 2.5, 0).tolist(), pdhi=np.percentile(MD_, 97.5, 0).tolist(),
           pelo=np.percentile(ME_, 2.5, 0).tolist(), pehi=np.percentile(ME_, 97.5, 0).tolist())
json.dump(out, open("johnson_neyman.json", "w"))
sig = np.array(out["pdhi"]) < 0
print(f"  difficulties route supported to approximately {weeks[sig].max():.0f} weeks")
print("  Written: johnson_neyman.json (input to 07_figures.py)")
```

File S1 source listing: 04_sensitivity_antibody.py

```
"""04 — Table S2: the antibody specification set (seven codings) and stratified re-estimation."""
import numpy as np
from core import load, bootstrap, fit_modmed, fit_mediation, show, COLUMNS, TPO_LLOQ, TPO_CUTOFF

d, covm = load(); C = COLUMNS
t = d[C["tpo"]].values
zz = lambda v: (v - np.mean(v)) / np.std(v, ddof=1)

codings = [
    ("Binary, >= 60 U/mL (manufacturer, primary)", (t >= 60).astype(float)),
    ("Binary, >= 34 U/mL (legacy coding)",         (t >= 34).astype(float)),
    ("Continuous, natural log, as reported",       np.log(t)),
    ("Continuous, values < 28 set to 28",          np.log(np.where(t < TPO_LLOQ, 28.0, t))),
    ("Continuous, values < 28 set to 14",          np.log(np.where(t < TPO_LLOQ, 14.0, t))),
    ("Continuous, values < 28 set to 19.8",        np.log(np.where(t < TPO_LLOQ, 19.8, t))),
    ("Ordered three-level (<28 / 28-59 / >=60)",   np.select([t < 28, (t >= 28) & (t < 60), t >= 60],
                                                             [0, 1, 2]).astype(float)),
]
print("=== Index of moderated mediation under each coding of the antibody variable ===")
for label, v in codings:
    d["_w"] = zz(v)
    r = bootstrap(d, covm, fit_modmed, exposure="z_fT4", moderator="_w")
    print(f"\n{label}")
    show("  ERSQ skills route", r["idx_ersq"])
    show("  DERS-16 difficulties route", r["idx_ders"])
    show("  Difference", r["idx_diff"])

print("\n=== Stratified re-estimation of the primary models ===")
for lab, m in [(f"anti-TPO <  {TPO_LLOQ:.0f} U/mL", t < TPO_LLOQ),
               (f"anti-TPO >= {TPO_LLOQ:.0f} U/mL", t >= TPO_LLOQ),
               (f"anti-TPO <  {TPO_CUTOFF:.0f} U/mL", t < TPO_CUTOFF),
               (f"anti-TPO >= {TPO_CUTOFF:.0f} U/mL", t >= TPO_CUTOFF)]:
    r = bootstrap(d, covm, fit_mediation, mask=m, exposure="z_fT4")
    show(f"{lab} (n = {r['total']['n']}) — total", r["total"])
    show(f"{lab} (n = {r['total']['n']}) — indirect", r["ind_total"])

print("\n=== Exclusion sensitivity analyses (Reviewer 2, comment 8) ===")
for lab, m in [("Excluding gestational diabetes", (d.gestational_diabetes == 0).values),
               ("Excluding unplanned pregnancy", (d.planned_pregnancy == 1).values),
               ("Excluding both", ((d.gestational_diabetes == 0) & (d.planned_pregnancy == 1)).values)]:
    r = bootstrap(d, covm, fit_mediation, mask=m, exposure="z_fT4")
    show(f"{lab} (n = {r['total']['n']}) — total", r["total"])
    show(f"{lab} (n = {r['total']['n']}) — indirect", r["ind_total"])
```

File S1 source listing: 05_descriptive_tables.py

```
"""05 — Tables 1-3."""
import numpy as np, pandas as pd
from scipy import stats
from core import load, COLUMNS

d, _ = load(); C = COLUMNS; N = len(d)
print("=== Table 1 — sample characteristics ===")
for col, lab in [("age_years", "Age, years"), (C["ga"], "Gestational age, weeks"),
                 ("prepregnancy_bmi", "Pre-pregnancy BMI, kg/m2"), (C["tsh"], "TSH, mIU/L"),
                 (C["ft4"], "Free T4, ng/dL"), (C["ft3"], "Free T3, pg/mL"),
                 (C["ders"], "DERS-16 total"), (C["ersq"], "ERSQ total")]:
    v = d[col]
    print(f"  {lab:28s} mean {v.mean():7.3f} (SD {v.std(ddof=1):6.3f})   median {v.median():7.3f}")

print("\n=== Table 2 — biochemical phenotype (mutually exclusive, exhaustive) ===")
tsh, ft4 = d[C["tsh"]], d[C["ft4"]]
lt4 = d[C["lt4"]] == 1
band = pd.cut(tsh, [-np.inf, 0.55, 4.0, np.inf], right=False, labels=["< 0.55", "0.55-3.99", ">= 4.00"])
low = ft4 < 0.89
total = 0
print(f"  {'LT4':5s} {'TSH band':11s} {'fT4 low':>8s} {'fT4 normal':>11s} {'Total':>7s} {'%':>7s}")
for tr, mask in [("Yes", lt4), ("No", ~lt4)]:
    for b in ["< 0.55", "0.55-3.99", ">= 4.00"]:
        m = mask & (band == b)
        a, c = int((m & low).sum()), int((m & ~low).sum())
        total += a + c
        print(f"  {tr:5s} {b:11s} {a:8d} {c:11d} {a+c:7d} {100*(a+c)/N:6.1f}%")
assert total == N, "the cells must partition the cohort exactly once"
print(f"  TOTAL {total} — partition verified")

print("\n=== Table 3 — measures by trimester ===")
d["tri"] = pd.cut(d[C["ga"]], [-np.inf, 13.99, 27.99, np.inf], labels=["T1", "T2", "T3"])
for col in [C["tsh"], C["ft4"], C["ft3"], C["ders"], C["ersq"], C["dist"]]:
    g = [d.loc[d.tri == k, col].values for k in ["T1", "T2", "T3"]]
    F, pf = stats.f_oneway(*g)
    H, ph = stats.kruskal(*g)
    print(f"  {col:22s} " + "  ".join(f"{x.mean():6.2f} ({x.std(ddof=1):5.2f})" for x in g)
          + f"   ANOVA p = {pf:.3f}   Kruskal-Wallis p = {ph:.3f}")
```

File S1 source listing: 06_precision.py

```
"""06 — Section 3.6 and Table S3: precision of the design.

Retrospective observed power is deliberately NOT computed: at a fixed sample size it is a
monotone transformation of the p value and conveys no information beyond the reported
confidence intervals.
"""
import numpy as np
from scipy import stats

half = lambda lo, hi: (hi - lo) / 2
print("=== Confidence-interval half-widths (from Tables 4 and 5) ===")
rows = [("Total indirect, log TSH", 0.141, 0.093, 0.192),
        ("Total indirect, free T4", -0.146, -0.199, -0.098),
        ("Index, gestational week x DERS-16", 0.047, 0.018, 0.078),
        ("Index, antibody x ERSQ", -0.027, -0.052, -0.007)]
for lab, est, lo, hi in rows:
    h = half(lo, hi)
    print(f"  {lab:38s} half-width {h:.3f}   ({100*h/abs(est):.0f}% of the point estimate)")

est, lo, hi = -0.027, -0.052, -0.007
se = half(lo, hi) / 1.96
mde = (stats.norm.ppf(0.975) + stats.norm.ppf(0.80)) * se
n_req = 360 * (mde / abs(est)) ** 2
print("\n=== Design analysis ===")
print(f"  standard error implied by the antibody-route interval : {se:.4f}")
print(f"  minimum detectable index at 80% power, alpha = 0.05    : {mde:.3f}")
print(f"  sample size required to detect an index of {abs(est):.3f}     : {n_req:.0f}")
```

File S1 source listing: 07_figures.py

```
"""07 — Figures 1-3 at 600 dpi. Run 03_moderation.py first (it writes johnson_neyman.json)."""
import json
import matplotlib; matplotlib.use("Agg")
import matplotlib.pyplot as plt
import numpy as np
from core import load, COLUMNS, z

plt.rcParams.update({"font.family": "DejaVu Sans", "font.size": 8, "axes.linewidth": .7,
                     "savefig.dpi": 600, "axes.spines.top": False, "axes.spines.right": False,
                     "figure.facecolor": "white"})
INK, A, B, GA, GB = "#1b1b1b", "#2F6F9F", "#B4643C", "#9FBFD8", "#E0BFAC"
d, _ = load(); C = COLUMNS

def save(fig, name):
    fig.savefig(f"{name}.png", dpi=600, bbox_inches="tight")
    fig.savefig(f"{name}.tif", dpi=600, bbox_inches="tight", pil_kwargs={"compression": "tiff_lzw"})
    plt.close(fig)

# Figure 1 — STROBE flow
fig, ax = plt.subplots(figsize=(6.69, 5.2)); ax.axis("off")
for x, y, w, h, t in [(.30,.88,.40,.07,"Screened for eligibility\nn = 452"),
                      (.30,.72,.40,.07,"Eligible\nn = 398"),
                      (.30,.56,.40,.07,"Enrolled\nn = 371"),
                      (.30,.36,.40,.09,"Analysed\nn = 360 (90.5% of eligible)\nno missing data on analysed variables")]:
    ax.add_patch(plt.Rectangle((x, y), w, h, fc="white", ec=INK, lw=.9))
    ax.text(x+w/2, y+h/2, t, ha="center", va="center", color=INK, fontsize=8)
for x, y, w, h, t in [(.72,.79,.27,.10,"Not eligible (n = 54)\nmultiple gestation 13 - major fetal anomaly 8\nGraves disease/thyrotoxicosis 5\npsychotic or bipolar disorder / psychotropic\ntherapy
11 - acute hospitalisation 7\nunable to complete questionnaires 6\nage < 18 years 1 - duplicate record 3"),
                      (.72,.635,.27,.05,"Declined participation\nn = 27"),
                      (.72,.465,.27,.06,"Excluded after enrolment (n = 11)\nincomplete questionnaire set or\nsame-visit biochemistry not linkable")]:
    ax.add_patch(plt.Rectangle((x, y), w, h, fc="#F5F5F5", ec="#8a8a8a", lw=.6))
    ax.text(x+.008, y+h/2, t, ha="left", va="center", color=INK, fontsize=6.0)
for y0, y1 in [(.88,.79),(.72,.63),(.56,.45)]:
    ax.annotate("", xy=(.5,y1), xytext=(.5,y0), arrowprops=dict(arrowstyle="-|>", color=INK, lw=.9))
for y in [.835,.675,.515]:
    ax.annotate("", xy=(.72,y), xytext=(.5,y), arrowprops=dict(arrowstyle="-|>", color="#8a8a8a", lw=.7))
ax.set_xlim(0,1); ax.set_ylim(.30,.98); save(fig, "Figure1")

# Figure 2 — exposure vs distress
fig, axes = plt.subplots(1, 2, figsize=(6.69, 2.9), sharey=True)
for ax, (v, lab, col) in zip(axes, [(z(np.log(d[C["tsh"]])), "Standardized log TSH", A),
                                    (z(d[C["ft4"]]), "Standardized free T4", B)]):
    y = d[C["dist"]].values
    ax.scatter(v, y, s=7, alpha=.45, color=col, edgecolors="none")
    b = np.polyfit(v, y, 1); xs = np.linspace(v.min(), v.max(), 100); r = np.corrcoef(v, y)[0, 1]
    se = np.std(y - np.polyval(b, v), ddof=2) * np.sqrt(1/len(v) + (xs-v.mean())**2/np.sum((v-v.mean())**2))
    ax.fill_between(xs, np.polyval(b, xs)-1.96*se, np.polyval(b, xs)+1.96*se, color=col, alpha=.16, lw=0)
    ax.plot(xs, np.polyval(b, xs), color=col, lw=1.4); ax.axhline(0, color="#c8c8c8", lw=.6, zorder=0)
    ax.set_xlabel(lab + " (z-score)")
    ax.text(.03, .95, f"r = {r:+.2f}", transform=ax.transAxes, va="top", fontsize=7.5, color=INK)
axes[0].set_ylabel("Antenatal distress composite\n(mean of four z-scores)")
axes[0].text(-.18, 1.06, "(a)", transform=axes[0].transAxes, fontweight="bold", fontsize=9)
axes[1].text(-.08, 1.06, "(b)", transform=axes[1].transAxes, fontweight="bold", fontsize=9)
fig.tight_layout(); save(fig, "Figure2")

# Figure 3 — two-panel Johnson-Neyman
j = json.load(open("johnson_neyman.json")); w = np.array(j["weeks"])
fig, axes = plt.subplots(1, 2, figsize=(6.69, 3.15), sharey=True)
for ax, (pt, lo, hi, col, gcol, ttl, shade) in zip(axes, [
        (np.array(j["pd"]), np.array(j["pdlo"]), np.array(j["pdhi"]), A, GA, "(a) Difficulties route (DERS-16)", True),
        (np.array(j["pe"]), np.array(j["pelo"]), np.array(j["pehi"]), B, GB, "(b) Skills route (ERSQ)", False)]):
    ax.fill_between(w, lo, hi, color=gcol, alpha=.8, lw=0, label="bootstrap 95% CI")
    ax.plot(w, pt, color=col, lw=1.6, label="conditional indirect association")
    ax.axhline(0, color=INK, lw=.8)
    sig = hi < 0
    if sig.any():
```

```
        bnd = w[sig].max()
        if shade: ax.axvspan(30.5, 38.5, color="#8a8a8a", alpha=.10, lw=0)
        ax.axvline(bnd, color="#6b6b6b", lw=.9, ls=(0, (4, 3)))
        lab = (f"supported to ~{bnd:.0f} wk\n(bootstrap 95% CI for the\nboundary: 30-38 wk)" if shade
               else f"supported to ~{bnd:.0f} wk")
        ax.annotate(lab, xy=(bnd, .045), xytext=(9, .045), fontsize=6.3, color="#3a3a3a", va="top")
    ax.set_xlabel("Gestational week"); ax.set_title(ttl, fontsize=8, loc="left"); ax.set_xlim(7, 39)
axes[0].set_ylim(-0.25, 0.10)
axes[0].set_ylabel("Conditional indirect association\n(free T4 exposure)")
h, l = axes[0].get_legend_handles_labels()
fig.legend(h, l, frameon=False, fontsize=6.8, ncol=2, loc="lower center", bbox_to_anchor=(.5, -.04))
fig.tight_layout(rect=[0, .05, 1, 1]); save(fig, "Figure3")
print("Figures 1-3 written at 600 dpi (PNG and TIFF).")
```
